# NS5-targeting nucleoside analogs inhibit dengue virus and other flaviviruses

**DOI:** 10.1371/journal.ppat.1013970

**Published:** 2026-02-17

**Authors:** Priyanka Bhakt, Swechha M. Pokharel, Yue Li, Tamanna Srivastava, Jesse Miller, Mark Dittmar, Yongqing Zhu, David Nguyen, Zachary Walter, Kasirajan Ayyanathan, Matthew Tudor, Chenguang Yu, Arnab K. Chatterjee, Holly Ramage, David Schultz, Sara Cherry

**Affiliations:** 1 Department of Pathology and Laboratory Medicine, University of Pennsylvania, Philadelphia, Pennsylvania, United States of America; 2 Department of Biochemistry and Biophysics, High throughput screening core, University of Pennsylvania, Philadelphia, Pennsylvania, United States of America; 3 Department of Microbiology and Immunology, Thomas Jefferson University, Philadelphia, Pennsylvania, United States of America; 4 Department of Cell and Developmental Biology, University of Pennsylvania, Philadelphia, Pennsylvania, United States of America; 5 Calibr-Skaggs at Scripps Research Institute, La Jolla, California, United States of America; Duke-National University of Singapore, SINGAPORE

## Abstract

Dengue virus (DENV) is a mosquito-transmitted flavivirus that circulates globally as four distinct serotypes and poses a substantial threat to public health. There are an estimated ~96 million symptomatic infections yearly, including severe cases of dengue fever, underscoring the urgency of identifying effective therapeutics targeting all four serotypes. Nucleoside analogs, which mimic endogenous nucleosides to inhibit viral RNA replication, offer a promising strategy for broad-spectrum antiviral development. Here, we conducted a high-throughput screen of 1,101 nucleoside analogs against DENV serotype 2 (DENV2) in a panel of human cell models, including human epithelial cells, hepatocytes, and fibroblasts. Candidates that were active against DENV2 were screened against all four serotypes. Since flaviviruses including West Nile virus and Zika virus are also important human pathogens, we screened these compounds for activity and identified compounds that were broadly active in these cellular and viral models. We further evaluated antivirals in primary human keratinocytes and fibroblasts, which are early targets of mosquito-transmitted DENV infection. From this screen, we identified 23 nucleoside analogs with broad antiviral activity against DENV and focused on two purine analogs UPGNUC255 and UPGNUC558, that demonstrated potent pan-flaviviral activity achieving >10-fold viral load reduction across all four DENV serotypes and other flaviviruses across cell models. Mechanistic studies revealed that both compounds target the viral RNA-dependent RNA polymerase (RdRp) domain of NS5. Resistance to UPGNUC558 was associated with a conserved S604T substitution, conferring cross-resistance to other 2′C-substituted nucleoside analogs. Resistance to UPGNUC255 was linked to a previously unknown R355Q mutation, located near the catalytic GDD motif of RdRp. These findings highlight UPGNUC255 and UPGNUC558 as promising leads for the development of broad-spectrum antiviral agents against flaviviruses.

## Authors summary

Dengue virus (DENV) infects millions of people each year, yet no specific antiviral treatment is available. Nucleoside analogs are a broad class of potentially broad-spectrum antivirals that mimic natural RNA building blocks. Therefore, we screened over a thousand nucleoside analogs, to identify inhibitors that are effective across cell types and against all four serotypes of DENV as well as related flaviviruses. We discovered two purine analogs, UPGNUC255 and UPGNUC558, that potently blocked replication of all four DENV serotypes and other flaviviruses including West Nile virus and Zika virus. These compounds target the viral RNA polymerase, a key enzyme required for viral RNA synthesis, as mutations near the catalytic site of this enzyme alter sensitivity. Our findings uncover promising broad-spectrum antiviral candidates and valuable insights into mechanisms of resistance among flaviviruses paving the way for next-generation broad-spectrum antivirals.

## Introduction

Dengue is a mosquito-borne virus that poses a threat and economic burden to many tropical and subtropical regions. It has become a major global health concern due to its recent expansion into non-endemic areas, including parts of the United States and Europe [1–3]. It is estimated that there are between 100 and 400 million dengue infections annually [4]. According to the latest global dengue surveillance data from the World Health Organization, since the start of 2024, approximately 13 million cases of dengue have been reported worldwide, including nearly 42,000 classified as severe and more than 8,700 deaths [5]. Over the past three decades, the transmission of dengue virus and the prevalence of its primary vector, *Aedes aegypti* mosquitoes, have expanded largely due to factors such as climate change, population growth, increased travel, and rapid urbanization [6–9]. Dengue infection is caused by four antigenically distinct but genetically related serotypes (DENV1–4) with a disease spectrum that can range from asymptomatic or mild febrile illness in most cases to severe life-threatening conditions, such as hemorrhagic fever and shock syndrome. These severe manifestations are often associated with plasma leakage and low blood pressure, which can be fatal if not managed promptly. [8,10,11] The risk associated with each serotype varies depending on the timing and sequence of infection. [12–14]. The only approved vaccines are Dengvaxia, which is restricted to individuals who are seropositive due to its limited efficacy across all four dengue serotypes, and Qdenga (TAK-003), which is effective against all four serotypes primarily in previously infected individuals. In dengue-naïve individuals, its efficacy is limited mainly to DENV-1 and DENV-2 [15]. This underscores the need for additional treatments.

DENV belongs to the viral family *Flaviviridae*, and genus *Orthoflavivirus*, which are arthropod-borne, enveloped, spherical viruses containing positive-sense, single-stranded RNA genomes. In addition to DENV, this family includes Zika virus (ZIKV), West Nile virus (WNV), Japanese encephalitis virus (JEV) and Yellow fever virus (YFV), all of which are widespread and of medical significance [16,17]. ZIKV is primarily transmitted by *Aedes* mosquitoes but can also spread through sexual contact, blood transfusion, and from mother to fetus causing Congenital Zika Syndrome [18–20]. In 2016, the World Health Organization declared ZIKV a Public Health Emergency of International Concern due to its rapid spread and its status as the first major infectious disease linked to birth defects [20,21]. WNV is a globally distributed mosquito-borne flavivirus and a leading cause of viral encephalitis resulting in over 24,000 neuroinvasive cases and approximately 2,300 deaths in the United States since its introduction in 1999 [22]. While most infections of WNV are asymptomatic, < 1% develop severe neurologic disease. In contrast, Kunjin virus (KUNV), a subtype of West Nile virus (WNV) found in Australia, is less pathogenic and exhibits reduced virulence in humans [23,24]. JEV is the leading cause of viral encephalitis in Asia. Most infections are asymptomatic or mild, but about 1 in 250 cases develop severe neuroinvasive disease, often resulting in neurological damage or death. There is no specific antiviral treatment, but the WHO-prequalified live attenuated SA14–14–2 vaccine is widely used for prevention in endemic areas [25,26]. YFV is a mosquito-borne flavivirus with high mortality causing a fatal hemorrhagic disease characterized by jaundice from liver injury. YFV is endemic in both Africa and South America. Although a highly effective live attenuated vaccine is available, yellow fever continues to pose a significant public health risk due to low vaccination coverage [27,28].

Flaviviruses are transmitted to humans by mosquitoes during a bloodmeal; many infecting and replicating locally in the skin, including keratinocytes, fibroblasts, dendritic cells and macrophages [29,30]. Migration of infected cells to lymph nodes facilitates subsequent dissemination of flaviviruses through blood stream which can infect additional secondary organs including the liver for DENV and YFV [31,32].

All RNA viruses replicate using an RNA-dependent RNA polymerase (RdRp) which is encoded by NS5 in flaviviruses. Viral RdRps are highly conserved, and thus antivirals that target this conserved enzyme can have activity against multiple flaviviruses allowing the potential development of broad-spectrum inhibitors [33]. Viral RdRps can incorporate nucleoside analogs which can inhibit viral RNA production [34]. Upon incorporation into the nascent viral RNA, nucleoside analogs can introduce mutations or lead to the premature termination of RNA synthesis thereby inhibiting viral replication downstream [35–38]. The goal is to identify nucleoside analogs that are readily incorporated by viral RdRp, but not host polymerases [39,40]. Those that are also incorporated by cellular polymerases can show cellular toxicity [41–43].

Several nucleoside analogs have been evaluated in clinical or preclinical trials for their potential to treat dengue. Balapiravir (R1626) is a cytidine analog, that was first developed for the *Flaviviridae* member hepatitis C virus (HCV) [44]. Once the parent nucleoside is converted to its active triphosphate form by cellular enzymes and the triphosphate is incorporated by the DENV RNA-dependent RNA polymerase, leading to premature termination of viral RNA synthesis [45]. Balapiravir was shown to be active against diverse flaviviruses *in vitro* but in clinical trials it did not improve virological, immunological, or clinical outcomes when treatment was initiated within 48 hours of illness onset [46]. NITD-008 is a 2’-C-methyl adenosine analog that exhibited potent activity against dengue virus *in vitro* and in animal models [47,48]. NITD-008 causes premature termination of RNA synthesis by incorporating in viral RNA by targeting RdRp [48]. NITD-008 shows robust activity across flaviviruses [48–51]. Despite its promising preclinical efficacy, it was discontinued before reaching clinical trials due to toxicity concerns observed in animal studies [48,52]. MK-0608 is another 2’-C-methyl adenosine analog that targets the DENV RdRp to inhibit viral replication and is broad-spectrum [53,54]. Phase I clinical trials showed safety, but did not advance clinically [55–57]. AT-752 is a guanosine nucleotide analog prodrug with oral availability developed by Atea Pharmaceuticals and has entered Phase II trials for the treatment of Dengue. It also shows broad spectrum activity against other flaviviruses [58,59]. AT-9010, the active triphosphate of AT-752, causes immediate termination of viral RNA synthesis upon incorporation by RdRp. This represents non-obligate chain termination of viral RNA synthesis despite its 3′-OH group [59]. AT-527 (Bemnifosbuvir -hemisulfate salt), its epimer, has recently been repurposed for clinical evaluation against severe acute respiratory syndrome coronavirus 2 (SARS-CoV-2) and hepatitis C virus (HCV) [60–62]. In summary, while several nucleoside analogs have demonstrated potential against DENV, challenges have hindered their clinical development. Nevertheless, this class of antivirals continues to be an area of active research due to its potential for broad-spectrum activity.

Therefore, we set out to identify additional nucleoside analogs with activity against DENV. We tested multiple cellular models of infection to ensure robust evaluation of compound efficacy and cytotoxicity. Importantly, nucleoside analogs are prodrugs that are metabolized by host cell kinases into their triphosphate forms, allowing for utilization by the viral RdRp [63]. Furthermore, different cell types exhibit varying kinase activities, leading to different antiviral efficacy that can hinder development as was observed with Balapiravir. Given that DENV infects a variety of cell types *in vivo*, and due to the differential activation of nucleoside analogs, we screened this library of nucleoside analogs in multiple cellular models to identify those with activity across relevant models. This approach provides a more physiologically relevant assessment and improves the translational potential of the *in vitro* results. Furthermore, we explored activity across a spectrum of flaviviruses including DENV1–4, West Nile virus (WNV), Kunjin virus (KUNV), Zika virus (ZIKV), and Yellow Fever virus (YFV), to identify those that may show pan-antiflaviviral activity. In total, we identified 12 nucleosides that inhibited DENV2 across at least 2 cell types of which 3 nucleosides analogs inhibited all four serotypes, and 7 showed antiviral activity against additional flaviviruses.

Altogether, we focused on two potent purine nucleoside analogs. UPGNUC558 which is a 2’-C-methyl adenosine analog structurally similar to NITD-008 and MK-0608 [47,48] and UPGNUC255 that is structurally similar to tubercidin. Tubercidin has been previously shown to have anti-Dengue activity, but with high toxicity due to incorporation by cellular polymerases [64,65]. Our findings demonstrate that these two nucleoside analogs exhibit high selectivity indices and broad-spectrum antiviral activity against medically important flaviviruses across diverse cell models. We further investigated the mechanisms of action of these candidates using the non-pathogenic Kunjin virus and identified resistant strains. Sequencing revealed residues important for nucleoside activity. As expected, KUNV resistant to UPGNUC558 selection revealed a S604T mutation, a conserved residue known to confer resistance to NITD-008 and MK-0608 [47,66]. Furthermore, as expected, UPGNUC558-resistant strains were cross-resistant to other 2’-C-methyl adenosine analogs consistent with a shared mode of inhibition targeting the viral polymerase. In contrast, UPGNUC255 resistance was associated with a R355Q substitution in the RNA-dependent RNA polymerase (RdRp) domain of NS5. UPGNUC255-resistant viruses showed cross-resistance to tubercidin and tubercidin related analogs, demonstrating a shared interaction site. Given the structural differences between UPGNUC255 and UPGNUC558, there was no cross resistance between these resistant strains, further indicating distinct mechanisms of inhibition. These studies reveal that structurally related compounds may share cross-resistance profiles, others can retain efficacy, providing opportunities for combination therapies to minimize resistance development.

## Results

### High throughput screening of nucleoside analogs against DENV in different cell models

**Fig 1:**
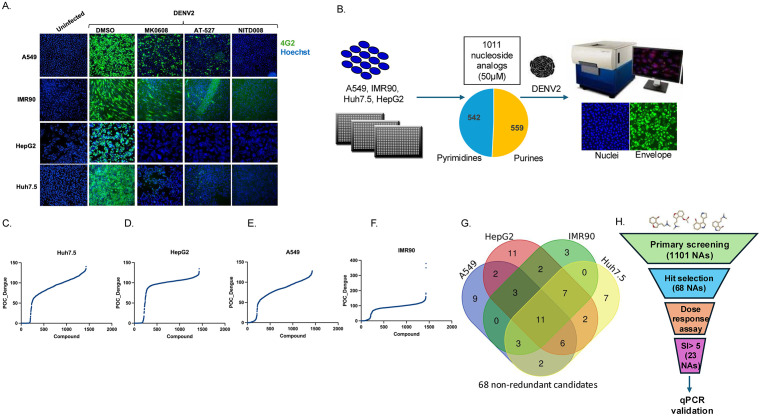
High throughput screening of nucleoside analogs identifies antivirals against DENV in different cell models: A. Representative fluorescence microscopy images of DENV2 infection in the indicated cell models (A549, IMR90, HepG2, and Huh7.5).Cells were treated with vehicle or the indicated compounds and either left uninfected or infected with DENV2 at MOI 2 (Huh7.5 and A549), MOI 1 (HepG2), and MOI 5 (IMR90). At 24 hours post-infection (hpi), cells were fixed and stained for viral antigen (4G2, green) and nuclei (Hoechst 33342, blue). Images were acquired at 10 × magnification. **B.** Schematic of the screening strategy. 384-well format high-throughput screen was conducted using A549, IMR90, HepG2, and Huh7.5 cells to evaluate a library of 1,101 nucleoside analogs for their ability to inhibit DENV2 infection at 50 μM. Cells were treated with compounds for 2 h then infected with DENV2 at the MOI in A, followed by fixation at 24 hours post-infection. Automated microscopy was used to quantify percent of infection and cell numbers normalized to vehicle control. Percent of control (POC) for DENV2 infection in **C.** Huh7.5 **D.** HepG2 cells **E.** A549 cells and **F.** IMR90 cells. **G.** Venn diagram representing 68 non-redundant candidates that showed >80 inhibition of infection; > 60% viability in at least one of the screened cell models **(C-F)**. **H.** Summary of hit selection and downstream validation showing the progression from 68 initial hits identified in four cell models to 23 candidates with SI > 5 based on dose–response analysis, followed by qPCR validation in selected cell models.

DENV targets diverse tissues *in vivo* and also can infect diverse cell types *in vitro* [18,67–69]. The liver is one of the most affected organs during Dengue hemorrhagic fever, and hepatocytes supports high levels of viral replication [70]. We developed assays in two liver cell models which includes human hepatocellular carcinoma Huh7.5 cells and, HepG2 cells, both of which have been previously used to study DENV infection [71,72]. Epithelial cells are also targets for DENV infection and thus we utilized human adenocarcinoma epithelial cells, A549 to model infection. Lastly fibroblasts are known targets of DENV infection, and we developed assays in non-transformed human lung fibroblast IMR90 cells. For each of these cell types, we optimized the infection conditions including viral MOI, and timing of infection using the prototype strain, New Guinea C of DENV 2 serotype (DENV2). We also optimized infection using previously known nucleoside analogs NITD-008 [48], MK-0608 [73] and AT-527 [59,74] shown to be active against DENV2. The antiviral activity of nucleoside candidates against Dengue was monitored using automated microscopy and immunostaining for DENV envelope (4G2) and nuclei (Fig 1A). Automated image analysis was used to quantify cell number and the percentage of infected cells compared to vehicle control. Dose response studies allowed us to quantify the 50% inhibitory concentration (IC_50_) and 50% cell toxicity concentration (CC_50_) for each nucleoside analog as well as the selectivity index (SI = CC_50_/IC_50_) (S1 Fig). We confirmed that all three known antivirals MK-0608, NITD-008 and AT-527 were active against DENV2 in all cell types tested (S1 Fig).

We screened an in-house nucleoside analog library which contains 1101 candidates, of which 559 are purines and 542 are pyrimidines at 50 μM. A schematic of the high-throughput screening is shown in Fig 1B. Cells were treated for 1 hour prior to infection and the cell number and percentage of infected cells were quantified 24 hours post infection using automated microscopy and image analysis in each cell model (Fig 1C-1F). Candidates with > 80% reduction in infection and with > 60% cell viability compared with DMSO control were selected in the primary screen. > 80% reduction in infection is a stringent cut-off to ensure that only compounds demonstrating strong antiviral efficacy were considered, similarly a ≥ 60% cell viability threshold (relative to the DMSO control) was applied to exclude compounds exhibiting excessive cytotoxicity that could confound the observed antiviral effect. The Venn diagram is shown for these compounds across the 4 different cell models. Under this threshold criteria, we identified a total 68 non-redundant candidates as active against DENV2 in at least one of the 4 cell models (Fig 1G **and** S1 Table). 11 compounds were identified across all four cell models and include nucleosides with known activity against DENV (Fig 1G **and** S1 Table).

### Defining nucleotide analogs with broad activity across different cell models

**Fig 2:**
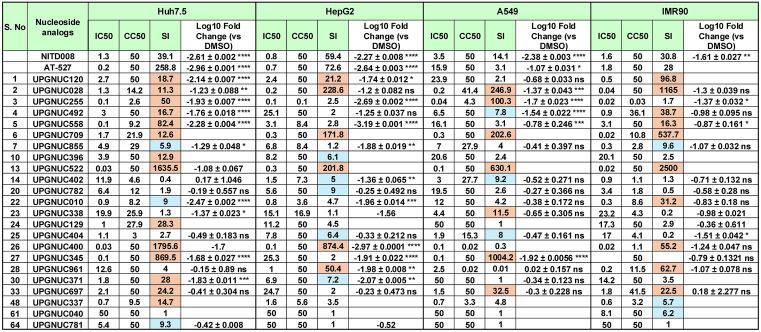
Dose response and orthogonal validation of nucleoside analogs: Table presents the comprehensive validation panel of IC_50_, CC_50_ (ATPlite), and SI values for indicated nucleoside analogs including the 23 primary hits from the DENV2 primary screen (n = 68) that exhibited a selectivity index (SI) > 5 in at least one cell model. The panel includes 2 positive controls (NITD-008 and AT-527). The serial numbers (S. no.) correspond to compounds listed in S1 Table. Blue SI > 5 and orange SI > 10. ATPlite data shown for each drug in each cell type at 72 h. SI shown for CC_50_ (ATPlite)/IC_50_. The log fold change from qPCR assessment in cells pretreated with the indicated compounds (10 μM) vs DMSO vehicle control infected DENV (MOI = 0.05) and subject to RT-qPCR 24 hours post-infection. Data are presented as mean ± SD, showing viral RNA levels relative to the vehicle control (n ≥ 1–3 independent biological replicates). Statistical significance was determined on n ≥ 3 by one-way ANOVA with Dunnett’s correction for multiple comparisons on log_10_-transformed values (*P < 0.05, **P < 0.01, ***P < 0.001, ****P < 0.0001).

We repurchased these 68 compounds and validated their activity against DENV2 using dose response assays (Fig 1H **and** S1 Table). We quantified infection using the same assay as the primary screen and we quantified toxicity outside of infection using ATPlite which monitors ATP levels as a surrogate for viability. While most compounds identified from the primary screening were evaluated in subsequent assays, not all compounds exhibiting activity in only one or two cell types were pursued for additional testing (S1 Table). From the tested candidates, 23 exhibited a SI greater than 5 in at least one cell model (Fig 1H **and** S1 Table). 5 had previously been reported to possess antiviral activity against flaviviruses, including Ribavirin [75], MK-0608 [53], 6-Azauridine, Azaribine (prodrug of 6-azauridine) [76] and azvudine [77]. 12 candidates were previously unstudied (Fig 2 **and** S1 Table).

We found that 14 compounds were 2’-C-methyl adenosine analogs, a modification known to confer antiviral activity (S6 Fig). This includes 2’-C-methyladenosine (UPGNUC129) [36], UPGNUC371 which resembles MK-0608 (UPGNUC120) structurally, and 2’-β-C-Ethynyladenosine (UPGNUC558) had not been previously shown to have antiviral activity against flaviviruses, and has similar features to NITD-008 (Figs 2, S2**, and** S3 **and** S1 Table). Azvudine (UPGNUC522) is another 2′-fluorinated nucleoside that has previously shown antiviral activity against HCV and DENV [77–79], and has recently demonstrated therapeutic promise against SARS-CoV-2 receiving conditional approval in China for the treatment of adult patients with COVID-19 [80]. UPGNUC345, a prodrug of Azvudine (UPGNUC522), contains a 2’-deoxy-2’-fluoro modification, a feature shared with UPGNUC125. Finally, gemcitabine (UPGNUC400), a widely used anticancer agent [81], serves as the parent nucleoside for UPGNUC961. Both compounds bear a 2’-deoxy-2’,2’-difluoro substitution and were found to be active against DENV2 in our screening (S6 Fig **and** S1 Table).

Tubercidin is a naturally occurring adenosine analog produced by *Streptomyces tubercidis*, and is known for its broad-spectrum antiviral activity. However, tubercidin’s therapeutic use is limited due to its high cytotoxicity in mammalian cells [64,65,82]. We identified 3’ deoxytubercidin (UPGNUC492), 6-Chloro-7-deazapurine-9-β-D-ribofuranose (UPGNUC255), and a prodrug for UPGNUC255 (UPGNUC855) which have structural similarity to tubercidin but have less cellular toxicity (S7 Fig).

Additionally, we identified several compounds including UPGNUC402, UPGNUC404, UPGNUC697, and UPGNUC782 that share structural similarities with N6-methyladenosine, which has been previously reported to exhibit antiviral effects against HIV, HBV, IAV, and other flaviviruses (S7 Fig) [83,84]. Among them, 6-chloroinosine (UPGNUC697) has been reported as both an antitumor and antiviral agent [85,86]. Another compound, UPGNUC010 (3’-Deoxy-3’-fluoroadenosine), has been reported to possess broad-spectrum antiviral activity against flaviviruses [87]. Lastly, UPGNUC740, UPGNUC338 and UPGNUC337 feature modifications at the 8’ carbon of the adenosine ring, while UPGNUC040 carries a hydroxyl substitution at the 8’ position (S7 Fig). Altogether, primary screen identified 68 initial hits of which 23 validated by dose–response analysis (S1 Table), confirming potency (IC_50_) and high SIs for the active compounds (Fig 2). A subset of these is active across multiple cell types suggesting efficient utilization by the nucleoside salvage pathways.

### Antiviral activity assessment by qPCR

To further quantify the antiviral activity of the identified compounds, we performed an orthogonal assay to quantify the reduction in DENV2 viral RNA across the cell models. Cells were pretreated with vehicle control (DMSO) or the indicated nucleoside analogs at 10 μM for 1hour and then infected with DENV2. NITD-008 and AT-527 were included as positive controls (Figs 2 **and** S3). As expected, both control compounds demonstrated >10-fold reduction in viral RNA levels across all tested cell types. The qPCR assays were performed for compounds showing a SI > 5 at 10 µM rather than 50 µM since it is challenging to achieve such high concentrations *in vivo* (S3A, S3B, S3C **and** S3D Fig). A subset that showed activity at 10 µM were also tested at 2 µM (S3E Fig). We also tested the prodrugs of UPGNUC255 (S3A, S3B, S3C **and** S3D Fig).

Consistent with results from our microscopy-based assay, UPGNUC255, and UPGNUC558, showed >10-fold reduction in DENV2 RNA in all four cellular models (Figs 2 **and** S3A-S3E). UPGNUC345 was active in Huh7.5, HepG2 and A549 whereas, UPGNUC028 and UPGNUC492 exhibited >10-fold reduction, but only in Huh7.5 and A549 cells. Additionally, UPGNUC855, UPGNUC010, UPGNUC338, UPGNUC371 and UPGNUC963 significantly reduced viral RNA levels in Huh7.5 and HepG2 cells. UPGNUC961 and UPGNUC402 demonstrated notable antiviral activity in HepG2 cells as well (Figs 2 **and** S3A-S3D). UPGNUC522 (Azvudine), a known antiviral agent, and UPGNUC400 (Gemcitabine), an anticancer drug with reported antiviral properties, both showed significant reductions in DENV2 RNA levels in all cell types tested (Figs 2 **and** S3A-S3D). Tubercidin-like compounds sharing structural features include UPGNUC255 and its prodrugs UPGNUC855 and UPGNUC963, as well as UPGNUC492, together with 2′-substituted analogues (UPGNUC558, and UPGNUC371), the 3′-modified compound UPGNUC010 (3′-deoxy-3′-fluoroadenosine), the azvudine-like analogue UPGNUC345, exhibited cell-type–dependent differences in antiviral potency. These variations likely arise from differences in cellular uptake, metabolic activation to the active triphosphate form, or host factor expression across cell models. Together, these findings highlight how specific structural modifications influence antiviral activity across distinct cellular environment and underscoring the role of structure–activity relationships in determining pan cell-type efficacy.

### Antiviral assessment across all four dengue serotypes

**Fig 3:**
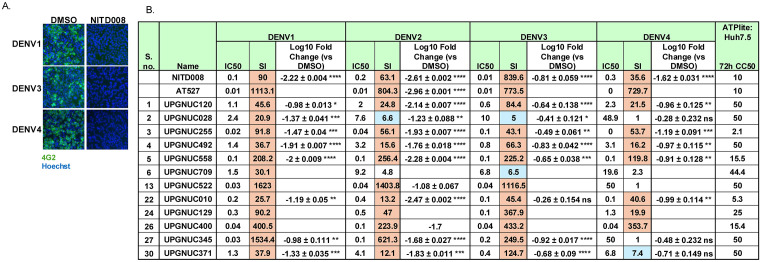
Nucleoside analog activity across DENV serotypes: A. Representative microscopy images for DENV1, DENV3 and DENV4 infection in the Huh7.5 cells were treated with either DMSO or NITD-008. Cells were fixed 24 hpi and stained for viral infection (4G2, green) and cell number (Hoechst 33342, blue). 10 × magnification. **B.** Table of IC_50_, CC_50_ (ATPlite), and SI values for selected drug candidates active against DENV2, evaluated in Huh7.5 cells infected with DENV1, DENV2, DENV3, or DENV4. Huh7.5 cells were infected with DENV1 (MOI = 1), DENV2 (MOI = 0.05), DENV3 (MOI = 1), or DENV4 (MOI = 0.5). The serial numbers (S. no.) correspond to in S1 Table. ATPlite data shown for each drug in each cell type at 72 **h.** SI shown for CC_50_ (ATPlite)/IC_50_ with blue SI > 5 and orange SI > 10. The log fold change from qRT-PCR analysis of dengue serotypes DENV1, DENV2, DENV3 and DENV4 infection for 24 h in Huh7.5 cells pretreated with the indicated compounds (10 μM) vs DMSO vehicle control. Data are presented as mean ± SD, showing viral RNA levels relative to the vehicle control (n ≥ 3 independent biological replicates). Statistical significance was determined for n ≥ 3 by one-way ANOVA with Dunnett’s correction for multiple comparisons on log_10_-transformed values (*P < 0.05, **P < 0.01, ***P < 0.001, ****P < 0.0001).

Dengue circulates in antigenically and genetically distinct serotypes (DENV1–4) [88]. These serotypes do not provide cross protection against each other and secondary infection with a distinct serotype can lead to more severe disease [89,90]. Therefore, it is essential for an antiviral to be effective against all 4 serotypes. We optimized DENV1, DENV3 and DENV4 infection conditions in Huh7.5 cells (Fig 3A) and performed dose response studies on the 12 compounds, which were found to be active for DENV2 confirmed antiviral potency (IC₅₀ ≤ 10 µM) confirmed by both dose response and qPCR assay and favorable selectivity index (SI > 5) in Huh7.5 cells from the primary screen to assess activity across dengue serotypes (Figs 2, S3A **and** S3B). Notably, all 12 compounds tested, exhibited a selectivity index (SI) > 5 against both DENV1 and DENV3, and 8 of these compounds showed an SI > 5 against DENV4, which is the genetically more distinct serotype (Fig 3B) [91,92].

We next quantified the antiviral activity of seven selected nucleoside analogs against all four DENV serotypes using RT-qPCR. These were more active candidates were chosen based on (i) strong pan-cell and pan-serotype inhibitory activity by dose response assay, (ii) structural diversity representing distinct nucleoside scaffolds, and (iii) consistent and robust inhibition of DENV2 replication in secondary qPCR validation assays. Notably, UPGNUC255, UPGNUC492, and UPGNUC558 reduced DENV1 RNA levels by >10-fold, DENV3 by >5-fold, and DENV4 by >10-fold in Huh7.5 cells, indicating broad-spectrum activity against multiple DENV serotypes (Figs 3B **and** S4A-S4C). In contrast, UPGNUC010 showed activity against DENV1 and DENV4, while UPGNUC028, UPGNUC345 and UPGNUC371 were active against DENV1 and DENV3 (Figs 3B **and** S4A-S4C). These results suggest that UPGNUC255, UPGNUC492, and UPGNUC558, reduced viral RNA levels across DENV1, DENV2, DENV3, and DENV4, highlighting their potential as pan-Dengue antiviral candidates. Moreover, compounds with serotype-specific activity, such as UPGNUC010, UPGNUC028, UPGNUC345, and UPGNUC371 may provide valuable insights into serotype-dependent mechanisms of antiviral efficacy. The pan DENV-serotype active compounds have broad-spectrum activity most likely results from targeting conserved viral replication mechanisms rather than serotype-specific or host-specific factors.

### Nucleotide analogs show broad activity against different flaviviruses

**Fig 4:**
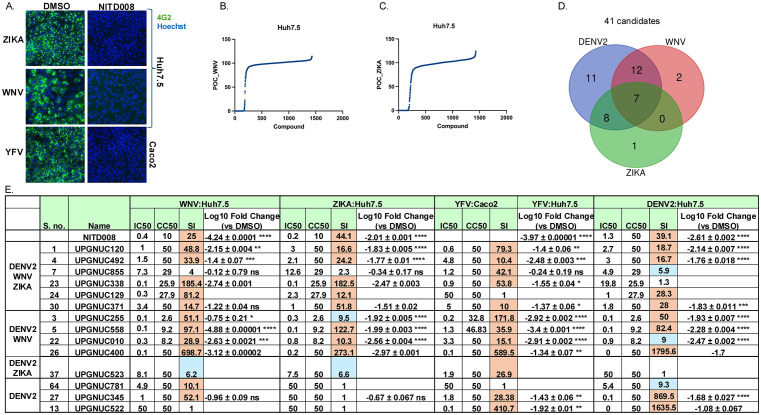
Identification of nucleoside analogs active across diverse flaviviruses: A. Representative microscopy images for Huh7.5 cells infected with WNV (NY2000) or ZIKA (MR766) and Caco-2 cells infected with YFV (17D) pretreated with either DMSO or NITD-008 (10uM). Cells were fixed 24 hpi and stained for viral infection (4G2, green) and cell number (Hoechst 33342, blue). 10 × magnification. Percent of control (POC) for (**B.**) WNV infection and (**C.**) ZIKA infection in Huh7.5 cells screened against the library of nucleoside analogs. **D.** Venn diagram showing 41 non-redundant candidate compounds with >80% inhibition of infection and >60% cell viability against DENV2, WNV or ZIKV, in Huh7.5 cells. **E.** Table of IC_50_, CC_50_ (ATPlite), and SI values for selected drug candidates against WNV, ZIKA, DENV2, infection in Huh7.5 cells and YFV in Caco2 cell with SI > 5 at least against one virus. The serial numbers (S. no.) correspond to S1 Table. ATPlite data shown for each drug in each cell type at 72 **h.** SI shown for CC_50_ (ATPlite)/IC_50_ with blue SI > 5 and orange SI > 10. The log fold change from qRT-PCR analysis of WNV infection; ZIKA infection YFV infection at MOI = 0.5 and DENV2 infection at MOI = 0.05 each in Huh7.5 cells pretreated with the indicated compounds (10 μM) or DMSO vehicle control at 24 hpi. qPCR data are presented as mean ± SD, showing viral RNA levels relative to the vehicle control (n ≥ 1-3 independent biological replicates). Statistical significance was determined for n ≥ 3 by one-way ANOVA with Dunnett’s correction for multiple comparisons on log_10_-transformed values (*P < 0.05, **P < 0.01, ***P < 0.001, ****P < 0.0001).

Next, we set out to identify the activity of nucleoside analogs against additional flaviviruses and thus we optimized HTS assays for WNV (NY2000) and ZIKV (MR766) in Huh7.5 cells, utilizing NITD-008 as a positive control (Fig 4A **and** S2 Table). We screened the in-house nucleoside analog library at 50 µM as we previously screened against DENV2 using a selection criteria of 80% reduction in viral infection and greater than 60% cell viability compared to the DMSO control (Fig 4B **and** 4C). We identified 21 compounds active against WNV and 16 compounds active against ZIKV (Fig 4D **and** S2 Table). Comparison with DENV2 screening data in Huh7.5 cells revealed 7 compounds that were active against all three viruses WNV, ZIKV, and DENV2. Additionally, 12 compounds were shared between DENV2 and WNV, and 8 compounds were common between DENV2 and ZIKV (Fig 4D **and** S2 Table). We performed dose-response assays for the compounds identified from the primary antiviral screens against WNV, ZIKV and which were active for DENV2 in Huh7.5 cells (S2 Table). We also optimized assays for YFV (vaccine strain17D) in Caco-2 cells using NITD-008 as a positive control (Fig 4A). We also tested the DENV2 active compounds against YFV and found a total of 20 compounds exhibited an SI > 5 against at least one additional flavivirus (S2 Table). Fourteen compounds showing activity against at least two flaviviruses with a selectivity index (SI) > 5 were included as the final panel for cross-flavivirus validation (Fig 4E).

We further quantified the activity of candidates from individual virus primary screens (S2 Table), including those active against DENV2, based on (i) strong antiviral activity across at least two flaviviruses by dose response assay (ii) consistent SI > 5, and (iii) structural diversity using qPCR assays against WNV NY2000 (Figs 4E **and** S4D), WNV Kunjin (Figs 4E **and** S4E), two strains of ZIKV (Puerto Rico and MR766 (Figs 4E, S4F **and** S4G) and YFV 17D (Figs 4E **and** S4H). We found that in addition to the positive controls, UPGNUC255, UPGNUC492, UPGNUC558, UPGNUC010, UPGNUC338, and UPGNUC400 exhibited potent antiviral activity, resulting in >10-fold reductions in viral RNA across all tested flaviviruses (Figs 4E **and** S4D-S4H). This suggests a conserved mechanism-of-action either directly on RdRp or a host enzyme. Additionally, UPGNUC371 (structurally similar to UPGNUC558, a 2′-substituted analog) showed antiviral activity against both ZIKV and YFV, and UPGNUC345 exhibited activity specifically against WNV and YFV (Figs 4E **and** S4D-S4H). These findings demonstrate *in vitro* pan-antiviral activity for UPGNUC255, UPGNUC492, and UPGNUC558.

### Nucleoside analogs show activity in primary cells

**Fig 5:**
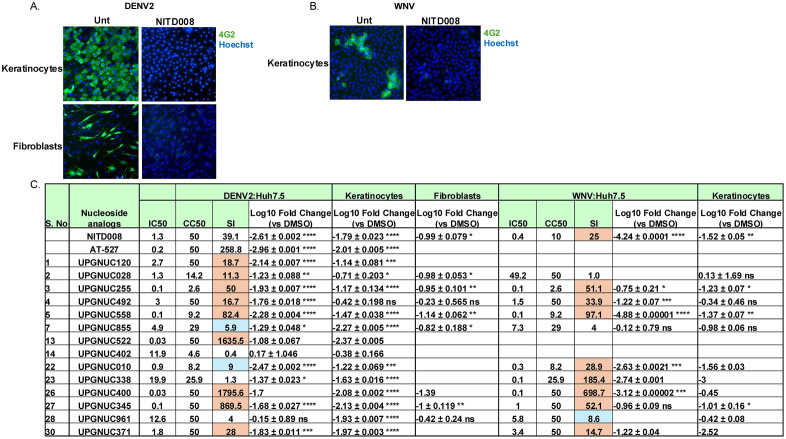
Nucleoside analog activity in primary cells: A. Representative fluorescence microscopy images of primary human keratinocytes and fibroblasts pretreated with either DMSO or 10 μM NITD-008 for 1 hour prior to infection with DENV2 (MOI = 1 for keratinocytes; MOI = 10 for fibroblasts). 48 hpi, cells were fixed and stained for viral antigen (4G2, green) and nuclei (Hoechst 33342, blue). Images captured at 10 × magnification. **B.** Representative fluorescence microscopy images of primary human keratinocytes pretreated with either DMSO or 10 μM NITD-008 for 1 hour prior to infection with WNV (MOI = 1). 48 hpi, cells were fixed and stained for viral antigen (4G2, green) and nuclei (Hoechst 33342, blue). Images captured at 10 × magnification. **C.** Table of IC_50_, CC_50_ (ATPlite), and SI values for selected drug candidates active against DENV2, and WNV evaluated in Huh7.5 cells. The log fold change from RT-qPCR assay in primary keratinocytes and primary fibroblasts pretreated with the indicated compounds (10 μM) vs DMSO vehicle control were infected with DENV2 (MOI = 0.5) or WNV (MOI = 0.5) for 48hpi. For all qPCR, data are presented as mean ± SD, showing viral RNA levels relative to the vehicle control (n ≥ 1-3 independent biological replicates). Statistical significance was determined for n ≥ 3 by one-way ANOVA with Dunnett’s correction for multiple comparisons on log_10_ transformed values (*P < 0.05, **P < 0.01, ***P < 0.001, ****P < 0.0001).

Our initial studies leveraged a panel of human cell lines including the cancer cell lines Huh7.5, HepG2, A549 and Caco-2 along with the immortalized but not transformed IMR90. We also set out to develop assays in primary human cells to confirm efficacy in more relevant cell types. During vector transmission, local infection at the skin is required [93,94]. Studies have shown that keratinocytes and fibroblasts are infected during flaviviruses infections; therefore, we tested antiviral activity in these primary cells. We optimized DENV2 infection protocols for primary human keratinocytes and fibroblasts and for WNV infection in primary human keratinocytes. We used NITD-008 as a positive control and found that treatment could block infection of DENV or WNV (Fig 5A **and** 5B). We also optimized a RT-qPCR assay again testing NITD-008, AT-527 and MK-0608 and found all three compounds resulted in approximately a 100-fold reduction in DENV2 viral RNA levels in primary human keratinocytes (Figs 5C **and** S5A). Additionally, treatment with NITD-008 achieved a ~ 100-fold decrease in West Nile virus (WNV) RNA levels in primary keratinocytes (Figs 5C **and** S5C). In primary fibroblasts, NITD-008 led to ~10-fold reduction in DENV2 RNA (Figs 5C **and** S5B).

Next, we evaluated the antiviral activity of selected nucleoside analogs from the validated panel chosen based on their activity against DENV2 in multiple cell lines and cross-flavivirus potency, specifically those showing (i) strong antiviral activity across at least two flaviviruses, (ii) consistent SI > 5, and (iii) structural diversity representing distinct chemical scaffolds, against DENV2 in primary keratinocytes using qPCR. In addition to the positive control drugs, UPGNUC028, UPGNUC255, UPGNUC558, UPGNUC855, UPGNUC522, UPGNUC010, UPGNUC338, UPGNUC400, UPGNUC345, UPGNUC961 and UPGNUC371, reduced DENV2 viral load by more than 10-fold (Figs 5C **and** S5A). Similarly, UPGNUC028, UPGNUC255, UPGNUC558, UPGNUC855, UPGNUC345 and UPGNUC400 reduced DENV2 infection 10-fold in primary fibroblasts (Figs 5C **and** S5B). In contrast, UPGNUC492 showed no significant reduction in viral load in primary keratinocytes and fibroblasts, indicating its lack of efficacy in primary cells (Figs 5C, S5A **and** S5B). We also tested activity of these nucleoside analogs against WNV and found that only UPGNUC255, UPGNUC558, UPGNUC345, UPGNUC010, UPGNUC371 and UPGNUC338 demonstrated more than a 10-fold reduction in viral in primary keratinocytes (Figs 5C **and** S5C), suggesting their potential as broad-spectrum antiviral candidates active in primary human cells.

UPGNUC492 possesses an unusual structural modification featuring a carbon substitution at the N-7 position of the purine ring and lacking the 3’-hydroxyl group on the ribose and exhibited low cytotoxicity in 3 models (Huh7.5, HepG2 and A549). However, its antiviral activity against DENV2 was only evident at higher concentrations across all four tested cell models (Huh7.5, A549, IMR90, and HepG2) by dose response assay, suggesting limited potency and making it a compound of reduced therapeutic interest (Figs 2, S2A **and** S2B). Similar to microscopy-based observations, RT-qPCR analysis confirmed significant viral RNA reduction at 10 µM only in Huh7.5 and A549 (Figs 2 **and** S3A-S3D), while no activity was observed at 2 µM (S3E Fig). Interestingly, UPGNUC492 showed broader antiviral activity, achieving ≥10-fold reductions in viral RNA levels for DENV1–4 (Figs 3C **and** S4A-S4C) and 500-, 10-, 100-, and 500-fold reductions in WNV, Kunjin, ZIKV, and YFV, respectively, at 10 µM tested in Huh7.5 cells (Figs 4E **and** S4D-S4H). Nevertheless, due to its lack of significant activity at lower concentrations, limited potency in key models with no activity in primary cells, UPGNUC492 is considered a less promising candidate.

### UPGNUC255 and UPGNUC558 are broadly active and potent across flaviviruses

Our data show that UPGNUC255 and UPGNUC558 display high potency and broad activity among different cell models including primary cells and were active against a panel of flaviviruses. UPGNUC558 is structurally related to NITD-008, distinguished by the presence of an ethynyl group at the 2’ position of the ribose moiety in the adenosine scaffold. UPGNUC558 shows activity across cell types against DENV2, and across all four DENV serotypes and broad-spectrum antiviral activity against WNV, KUNV, ZIKV, and YFV. Although both qPCR and microscopy assays showed UPGNUC558 and NITD-008 to have comparable efficacy in reducing viral RNA levels, UPGNUC558 consistently displayed lower IC₅₀ values and higher selectivity indices (SI) across multiple viruses, including DENV1, DENV2, DENV3, DENV4, WNV, KUNV, ZIKV, and YFV, in Huh7.5 cells (Figs 2, 3B **and** 4E). Given these observations, we set out to perform further mechanistic studies.

UPGNUC255, a synthetic adenosine nucleoside analog related to tubercidin (7-deazaadenosine). In addition to the substitution of the nitrogen at the purine N7 position with carbon, there is a replacement of the 6-amino group with a chlorine atom (S2A Fig). In our primary screen, Tubercidin (UPGNUC211, S1 Table) displayed activity which has been previously shown against diverse RNA viruses (e.g., poliovirus, rhinovirus, and Zika virus) but as expected, we also observed pronounced cytotoxicity (S1 Table) [65,82]. Although UPGNUC255 exhibited some cytotoxicity in IMR90 and HepG2 cells, it demonstrated a markedly improved selectivity index (SI) compared to tubercidin and structurally related analogs, primarily due to its higher antiviral potency at lower effective concentrations. UPGNUC255 exhibited broad-spectrum activity, significantly inhibiting replication of all four DENV serotypes, as well as WNV, Kunjin virus, ZIKV, and YFV (Figs 2, 3B**, and** 4E). These results highlight UPGNUC255 as a promising lead candidate, offering enhanced antiviral efficacy coupled with a more favorable therapeutic window than tubercidin.

We also identified two prodrugs of UPGNUC255, UPGNUC855 and UPGNUC963. UPGNUC855 displays reduced cytotoxicity but requires higher concentrations to achieve antiviral activity, as reflected in its elevated IC₅₀ values across all tested cell models (Fig 2) and reduced efficacy at 2uM (S3E Fig) and other flaviviruses (Figs 4E, S4D, S4H **and** S4G**).** In contrast, UPGNUC963 shows comparable IC₅₀ values to UPGNUC255 but exhibits greater cytotoxicity in hepatocytes and lung fibroblasts (S3A-S3D Fig **and** S1 Table), limiting its potential despite its antiviral potency.

We also synthesized two derivatives of UPGNUC255, mCot849 with a 4’ fluoro, and mCot903 with an additional chloro-group on the purine ring (S9 Fig). mCot849 exhibited antiviral potency comparable to UPGNUC255 (similar IC₅₀ values), along with a slightly improved cytotoxicity profile (higher CC₅₀), suggesting it may be a more favorable candidate than the parent compound. In contrast, mCot903 displayed higher IC₅₀ values compared to UPGNUC255, indicating reduced potency, and although it showed slightly improved cytotoxicity, its overall potential appears limited (S9 Fig).

### Effect of exogenous NTPs on antiviral activity of nucleoside analogs

**Fig 6:**
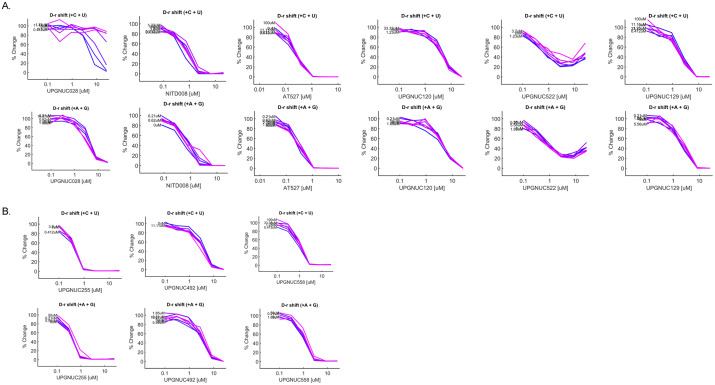
Nucleoside analog activity in the presence of exogenous nucleosides: Dose–response analysis of DENV2 infection of Huh7.5 cells with the reference (A.) and test (B.) nucleoside analogs in the presence of increasing concentrations of pyrimidine (C + U) or purine (A + G) nucleosides. At each concentration of the indicated nucleoside analogs (0.103, 0.309, 0.926, 2.778, 8.333, and 25 µM), corresponding concentrations of purine (A + G) were co-administered at 0.21, 0.62, 1.85, 5.56, 16.67, and 50 µM, respectively, and pyrimidine nucleosides (C + U) at 0.412, 1.23, 3.70, 11.11, 33.33, and 100 µM, respectively.

Direct acting nucleoside analogs compete with natural nucleoside triphosphates (NTPs) for binding to the RNA-dependent RNA polymerase (RdRP) for incorporation. Some compete efficiently while others compete less efficiently and require higher concentrations [95]. Furthermore, some nucleoside analogs can act by inhibiting the biosynthesis of natural NTPs, thereby inhibiting infection through this indirect mechanism and these can be readily competed with exogenous nucleosides [96]. Nucleoside analogs that can be readily competed are less likely to show activity *in vivo* in animals. To determine whether high levels of exogenous nucleosides can reduce the activity of our nucleoside analogs we treated cells with increasing concentrations of purines or pyrimidines in the presence of increasing doses of the nucleoside analogs. As a control, we tested 6-azauridine (UPGNUC028) which is phosphorylated to 6-azauridine-5’-phosphate to act as a competitive inhibitor of the enzyme orotidylate decarboxylase, required for pyrimidine biosynthesis [97,98]. Thus, 6-azauridine is known to inhibit *de novo* pyrimidine biosynthesis and should be competed with high levels of pyrimidines. In addition to its role as a *de novo* pyrimidine biosynthesis inhibitor, 6-azauridine can be phosphorylated to 6-azauridine triphosphate and incorporated into viral RNA, causing errors and leading to non-viable viral particles [99,100]. If 6-azauridine inhibited *de novo* biosynthesis as the mechanism of action we expected that treatment with exogenous pyrimidines would overcome the antiviral effects of 6-azauridine. Indeed, we observed dose-dependent decreases in antiviral activity of 6-azauridine when pyrimidines, but not purines, were added exogenously (Fig 6A). Next, we tested the other known antivirals NITD-008, AT-527, MK-0608, 2’-C-methyladenosine and Azvudine and observed no change in the dose response (d-r) curves upon addition of purines or pyrimidines (Fig 6A). Lastly, we tested our candidates UPGNUC255, UPGNUC492 and UPGNUC558 which were selected based on their novelty, broad antiviral activity across multiple cell models, and demonstrated efficacy against a panel of flaviviruses. These candidates represent the most active profiles for detailed mechanistic evaluation. Interestingly, we observed no significant change in the antiviral activity or shift in the dose response curves of these three nucleoside analogs by addition of either purines or pyrimidines (Fig 6B), suggesting that these nucleoside analogs are not targeting nucleoside biosynthesis pathways. Thus, these compounds are likely direct acting and potentially able to compete with endogenous nucleosides.

### UPGNUC255 and UPGNUC558 inhibit viral RNA replication through NS5

Nucleoside analogs can inhibit viral replication by direct incorporation into the active viral RNA template by the RdRp which can lead to chain termination or delayed chain termination (NITD-008, and AT-527) [48,101], stalling (Remdesivir) [102,103] or misincorporation (Molnupiravir) [104]. Others can impact additional RNA processing activities such as cap methylation (ribavirin and sinofungin) [105]. Since UPGNUC558 is similar to NITD-008 and MK-0608 we expect that the mechanism to inhibit viral RNA synthesis and that the amino acids required for antiviral activity would be similar across these analogs. In contrast, UPGNUC255 is quite distinct, suggesting that the requirements for incorporation may be distinct.

**Fig 7:**
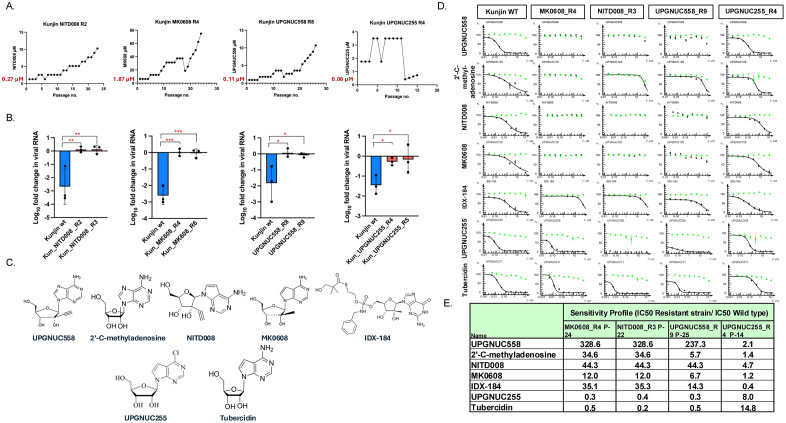
Resistance profiles to nucleoside analogs: A. Growth kinetics of KUNV strains amplified in Huh7.5 cells in the presence of increasing concentrations of the indicated nucleoside analogs. Red numbers = IC_50_s B. RT-qPCR analysis of KUNV resistant mutants (MOI = 0.5) in Huh7.5 cells pretreated with the indicated compounds (10 μM NITD-008, MK-0608, UPGNUC558; 2 uM UPGNUC255) or DMSO vehicle control 24hpi. Data are presented as mean ± SD, showing viral RNA levels relative to the vehicle control (n = 3 independent biological replicates). Statistical significance was determined by one-way ANOVA with Dunnett’s correction for multiple comparisons on log_10_-transformed values (*P < 0.05, **P < 0.01, ***P < 0.001). **C.** Chemical structures of nucleoside analogs related to UPGNUC558 and UPGNUC255. **D.** Dose response curves for Huh7.5 cells pretreated with the indicated drugs infected with the KUNV strains (MOI = 1) 24hpi with black showing POC infection and green showing POC cell viability. **E.** Table of the sensitivity profile of each selected KUNV strain compared to wild type for the indicated nucleoside analogs (IC_50_ Resistant strain/ Wild type) indicating the extent of cross-resistance to structurally diverse nucleoside inhibitors.

To identify the residues important for antiviral activity, we selected for viruses that were resistant to NITD-008, MK-0608, UPGNUC558 and UPGNUC255 by passaging viruses in the presence of gradually increasing concentrations of the compounds. Kunjin virus (KUNV), a non-pathogenic strain of West Nile virus (WNV), was used for resistance selection studies because it provides higher viral titers and exhibits more robust replication kinetics in cell culture, enabling faster generation and characterization of resistant variants. Importantly, the compounds selected for these experiments demonstrated potent antiviral activity against both KUNV and DENV, underscoring the broader relevance of the resistance findings across flaviviruses. We passaged the virus in the presence of each compound starting at IC_50_ concentration and DMSO was used as vehicle control across passages. Supernatants were harvested 2–3 days post infection at the onset of >50% CPE. Fig 7A shows the growth profiles of the KUNV resistant mutant selection. KUNV developed resistance to NITD-008, MK-0608, and UPGNUC558 after 22–26 passages (Fig 7A). We then expanded strains and compared the level of viral RNA replication in the wild type and resistant strains by RT-qPCR. We found that the resistant strains are no longer sensitive to the respective treatments (Fig 7B). Interestingly, resistance to UPGNUC255 began after 14 passages but only achieved a ~ 10-fold increase in EC₅₀ concentration and resistance to treatment as measured by RT-qPCR (Fig 7A **and** 7B).

We performed dose response studies to the selected nucleoside as well as a larger panel of nucleosides to determine if each strain resistant to the drug it was selected with was cross-resistant to other nucleosides. As expected by their similarity, we found that strains resistant to obtained against NITD-008, MK-0608 and UPGNUC558 exhibited similar patterns of cross-resistance, with more than 10-fold reduced susceptibility to 2’-C-methyl-modified nucleosides, including 2’-C-methyladenosine, 7-deaza-2’-C-methyladenosine (MK-0608), 7-deaza-2’-C-ethynyladenosine (NITD-008) and IDX184 (prodrug of 2′-methylguanosine) (Fig 7C-7E). These data indicate that these structurally related nucleoside analogs act through the same mechanism targeting the RdRp.

The KUNV strains resistant to UPGNUC255 displayed reduced sensitivity to UPGNUC255 and the related nucleoside analog tubercidin, with a ~ 14-fold increase in the IC_50_ compared to wild-type KUNV (Fig 7C-7E). In contrast, these resistant strains did not show markedly altered sensitivity to the 2’-C-methyl-modified nucleosides, suggesting specificity in the resistance phenotype (Fig 7D-7E).

### UPGNUC558 and UPGNUC255 target viral NS5 protein

**Fig 8:**
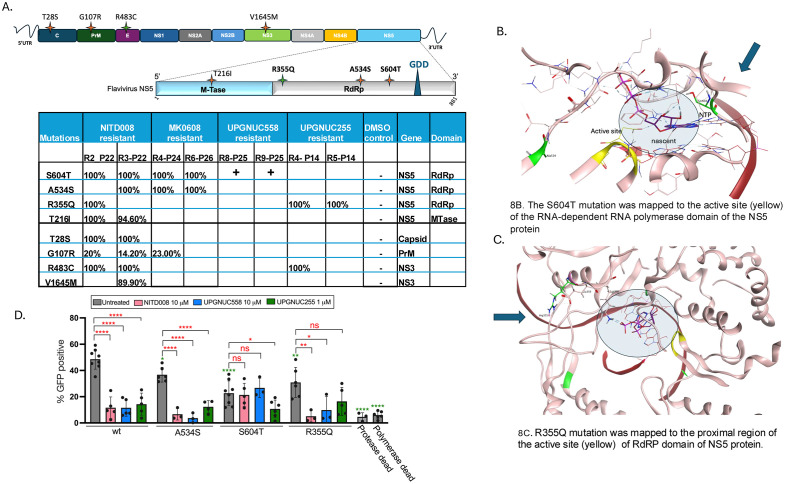
UPGNUC558 and UPGNUC255 interact with RdRp to confer antiviral activity: A. Sequencing of selected KUNV strains showing percent amino acid for each strain, gene and domain. “+” indicates that the mutation was identified by Sanger sequencing B. Modeling shows that the S604T substitution (green, with blue arrow) is localized near the active site (yellow) of the RNA-dependent RNA polymerase (RdRP) domain within the NS5 protein. The RNA template and incoming nucleoside in the primers site are also shown (grey circle). The A534 residue (green) is located far from the active site. **C.** Modeling shows that the R355Q mutation (green, blue arrow) is situated in the vicinity of the RdRP active site (yellow) of RdRp region of NS5. The RNA template is also shown with the incoming nucleoside (grey circle). **D.** Cells transfected with wild-type or specified alleles were treated with the nucleoside analogs as indicated. Mean±SD shown (n ≥ 3) and are normalized either to wild-type controls (indicated in green stars) or to the untreated condition of the respective samples (indicated in red stars). Statistical significance was determined using one-way ANOVA with Dunnett’s correction for multiple comparisons (*P < 0.05, **P < 0.01, ***P < 0.001, ****P < 0.0001).

To determine the amino acid changes that confer resistance we performed whole-genome sequencing (WGS) analysis on resistant mutants and DMSO selected control strains. WGS analysis of the resistant mutants revealed several single nucleotide changes, which were absent in the DMSO-treated control strain (Fig 8A). NITD-008 and MK-0608 are incorporated into the viral RNA, which inhibits RNA chain elongation [48,56]. Sequencing analysis found that all UPGNUC558-, NITD-008-, and MK-0608-resistant strains had a S604T mutation in the RdRp domain of the NS5 protein (Figs 8A and S10C). This mutation has been previously associated with resistance to 2’-C-methylated nucleoside analogues in flaviviruses, including WNV resistant to Sofosbuvir [106]; HCV resistant to 2’-C-methyl ribonucleosides and NITD-008 [47,101] and TBEV resistant to MK-0608 and 2’-C-methylcytidine [66]. This amino acid is conserved across flaviviruses (S10C Fig) and studies suggested that this Serine-to-Threonine substitution reduces analogue interactions and displaces metal ion cofactors [107]. Furthermore, the additional methyl group disrupts water-mediated hydrogen bonding with the 2’-C-methylated analogue, redirecting interactions towards substrate-binding regions [56,107].

In addition, we identified a previously unreported A534S mutation located within a conserved region of the NS5 RNA-dependent RNA polymerase (RdRP) domain (Figs 8A **and** S10B). This amino acid is conserved across flaviviruses (S10B Fig) and was present in one of the NITD-008-resistant mutants and in both MK-0608-resistant mutants. However, UPGNUC558 resistant strains did not encode this change.

The WGS analysis of UPGNUC255-resistant strains revealed a R355Q mutation in the conserved RdRp domain of NS5 (Figs 8A **and** S10A), which was present in both UPGNUC255-resistant strains (UPGNUC255 R4, R5 at passage 14) and in one NITD008-resistant strain (NITD008 R2). This amino acid is also conserved across flaviviruses (S10A Fig) and was not been previously reported among flaviviruses strains resistant to nucleoside analogs. We directly sequenced the residues around R355 and found that two additional strains resistant to UPGNUC255 also had this R355Q change (S10D Fig).

In order to visualize the location of the mutations in context, we modeled the Kunjin NS5 structure. The starting point was an AlphaFold 3 model of Dengue Type 2 NS5 in complex with an RNA template, growing strand, and incoming ATP nucleotide. A homology model of Kunjin was made based on this template and used to visualize the relative positions of the mutations with respect to the active site (GDD, yellow) and nucleic acids (Fig 8B-8C). Resistance-associated residues were mapped onto the RdRp domain of NS5 at positions S604, A534 (Fig 8B), and R355 (Fig 8C). The modeling revealed that the active nucleotide binds at the catalytic site, centered around the conserved GDD motif (yellow). Notably, residues S604 (green) and R355 (ball and stick) are located in close proximity to the active site, suggesting that they are gating the incoming nucleosides. In contrast, A534 (green) is positioned more distally and thus is less likely to be playing a role in nucleoside selection.

### Replicons confirm single amino acid changes confer resistance

To determine whether these amino acid changes confer resistance to the selected nucleosides we utilized a WNIIrep-GFP (WN 956 D117 3B) subgenomic replicon [108,109]. This subgenomic replicon encodes a genome capable of autonomous viral RNA replication but harbors a 5’ deletion of the structural genes C, prM and E, which are replaced with a GFP reporter. This replicon unable to generate infectious virions, but offers a sensitive approach to measure the impact of amino acid substitutions at specific residues on viral RNA replication [108,109].

As controls, we used the S135A mutant of NS3 and the D668A mutant of NS5 as these are replication-deficient strains. The S135A mutation renders the NS3 protease inactive, thereby inhibiting viral polyprotein processing, while the D668A mutation disrupts the RNA-dependent RNA polymerase (RdRp) activity of NS5, abrogating viral RNA synthesis [109]. We introduced individual A534S, S604T and R355Q mutations into the WNV replicon using site-directed mutagenesis. These mutant replicons were transfected into HEK293T cells to assess the impact of each mutation on viral replication in the presence and absence of nucleoside analogs, as measured by automated microscopy. First, we validated the antiviral activity of our nucleoside analogs in HEK293T cells against KUNV, observing a ≥ 10-fold reduction in viral RNA levels upon treatment with the compounds (S10E Fig). Upon transfection, the wild type (wt) replicon resulted in approximately, 50% of cells expressing the GFP reporter, whereas, polymerase dead (D668A) and protease dead (S135A) mutants exhibited more than 90% reduction in GFP expression compared to the wt replicon, confirming that GFP expression is dependent on RNA replication (Fig 8D). A534S, R355Q, and S604T each exhibited attenuated replication, reducing the percentage of cells expressing GFP by ~25%, 30% and 50%, respectively, compared to wt. This suggests that these mutations negatively impact viral fitness (Fig 8D).

Drug sensitivity assays revealed that treatment with NITD-008 and UPGNUC558 reduce wt and A534S more than 75% indicating that the A534S mutation does not confer resistance to these compounds (Fig 8D). In contrast, the S604T mutation abrogated antiviral activity of both NITD-008 and UPGNUC558, suggesting that this mutation confers resistance to these nucleoside analogs (Fig 8D).

As expected, the R355Q mutation did not alter sensitivity to NITD-008 or UPGNUC558 but conferred a moderate level of resistance to UPGNUC255. While UPGNUC255 effectively inhibited replication in wt replicon, it was significantly less effective against the R355Q mutant (Fig 8D). These results indicate that S604T confers resistance to both NITD-008 and UPGNUC558 while R355Q specifically impairs the efficacy of UPGNUC255. These findings provide critical insights into the antiviral mechanism of UPGNUC255, highlighting NS5 as a primary target and underscoring key resistance-associated mutations that cluster near the RdRp active site.

## Discussion

Ongoing changes in climate conditions have expanded the global distribution of areas that are environmentally suitable for vectors that transmit flaviviruses including those that transmit DENV [7,8]*.* The limitations of currently available vaccines and the absence of clinically approved antivirals for DENV underscore the urgent need to identify effective antiviral agents for both therapeutic treatment and prophylactic use against DENV. Nonstructural proteins in the flaviviruses are the major targets for antiviral development because of their enzymatic roles in viral replication. NS5 is one of the most conserved proteins encoded by flaviviruses with more than 75% sequence homology across all four DENV serotypes and a highly conserved pocket for RNA synthesis.

Nucleoside analogs can show high potency to inhibit viral replication, making them candidates for antiviral drug development. Toward the discovery of nucleoside analogs that inhibit DENV, we screened a nucleoside analog library. Since nucleoside analogs are prodrugs require host encoded enzymes for phosphorylation into the active triphosphate, there is often variable efficacy across cell types [110,111]. These include variability in the expression of nucleoside transporters that facilitate drug uptake, as well as enzymes required for the activation and metabolism of the compounds [112,113]. Additionally, the presence and activity of efflux transporters can modulate intracellular drug concentrations, while differences in cell proliferation rates may influence the extent of analog incorporation into endogenous nucleic acids [114,115]. By employing diverse cellular systems, we identify analogs with activity across cell types. [116,117]. Primary screening identified 68 nucleoside analogs with anti-DENV-2 activity in at least one of the four cell models. Of these, 11 were active in all four cell models. The majority of candidates were validated by dose–response assays having a SI > 5 in at least one model.

Our screening successfully identified several nucleoside analogs previously reported to have antiviral activity against flaviviruses, including MK-0608, 6-azauridine, azaribine, ribavirin, gemcitabine, and azvudine, thereby validating the robustness and sensitivity of our screening strategy.

We identified two novel purine analogs, UPGNUC255 and UPGNUC558, that exhibit potent antiviral activity against all four dengue virus serotypes across four distinct cell models, with low EC₅₀ values. Beyond their efficacy in cultured cell lines, both compounds also demonstrated significant antiviral effects in primary human keratinocytes and fibroblasts, underscoring their potential relevance in physiologically important systems. Moreover, these analogs displayed broad-spectrum activity against several medically important flaviviruses, including West Nile virus (WNV), Kunjin virus (KUNV), Zika virus (ZIKV), and yellow fever virus (YFV), supporting their promise as candidates for the development of pan-flaviviral therapeutic development.

UPGNUC558 and UPGNUC371, both featuring 2’ substitutions, are structurally related to established antiviral agents such as 2’-C-methyladenosine, MK-0608, IDX184 and NITD-008 compounds known for their efficacy against various flaviviruses. Previous studies on the mechanism of action of MK-0608, IDX184 and NITD-008 have shown that, once incorporated into the viral RNA, it prevents further elongation of the RNA chain, leading to premature termination of RNA synthesis and effectively halting viral replication [48,66,118]. Although, certain 2′-C-methyl–modified nucleoside analogues have failed in clinical development due to toxicity, these effects are largely compound-specific rather than inherently associated with the 2′-C-methyl modification itself since Sofosbuvir has shown great success. This modification remains a validated scaffold for viral RNA polymerase inhibition. The robust and consistent antiviral activity across multiple flaviviruses, including DENV, while maintaining favorable cytotoxicity profiles in both immortalized and primary human cells observed with compounds UPGNUC558 and UPGNUC371 underscore the potential of optimized 2′-C-methyl analogues with improved selectivity and metabolic stability for further development. In our study, we identified a shared resistance-associated residue, S604T in NS5, in Kunjin virus selected under pressure from UPGNUC558, NITD-008, and MK-0608, also show cross resistance with UPGNUC371 and 2’-C-methyladenosine. This residue has previously been linked to resistance against different nucleoside analogs in flaviviruses, underscoring its potential role in a conserved mechanism of antiviral resistance [54].

Tubercidin is a nucleoside analog that mimics adenosine and exhibits broad biological activity, including antiviral, antitumor, and antiparasitic effects. However, its application is limited due to its high cytotoxicity since tubercidin triphosphate can be incorporated into DNA or RNA by host polymerases [65]. In our primary screen, tubercidin exhibited antiviral activity close to the toxicity in most cell types (S1 Table). We identified a number of nucleosides related to tubercidin. UPGNUC492 is a 3′-deoxytubercidin analog previously reported as an anti-trypanosomal agent for the treatment of sleeping sickness, exhibited broad-spectrum antiviral activity against all four dengue serotypes as well as WNV, ZIKV, and YFV in our assays with lower toxicity than tubercidin. However, this compound was less active in primary human keratinocytes and fibroblasts.

UPGNUC255 differs from tubercidin by the substitution of the amine group with a chlorine atom at the 6′ position of the adenosine ring. UPGNUC255 demonstrated significantly lower cytotoxicity compared to tubercidin while retaining strong antiviral efficacy *in vitro*. We also identified two prodrugs of UPGNUC255, UPGNUC855 and UPGNUC963. Among these, UPGNUC855 exhibited broader antiviral activity, including efficacy in primary keratinocytes and fibroblasts, and showed lower cytotoxicity compared to UPGNUC963. However, unlike UPGNUC255, UPGNUC855 did not demonstrate activity against other flaviviruses such as West Nile virus (WNV) and Zika virus (ZIKV).

We also synthesized mCot849, which has a 4′ fluorine, and showed low cytotoxicity and an EC₅₀ comparable to that of UPGNUC255 against DENV2 infection in hepatocyte cells. Mechanism-of-action studies using nucleoside competition assays revealed that UPGNUC255 does not interfere with host nucleoside biosynthesis or salvage pathways, suggesting it acts as a direct-acting antiviral rather than targeting host nucleotide metabolism. We found that resistance to UPGNUC255 and its analogs was conferred by a single point mutation in the RdRp NS5: R355Q. This amino acid is in the index finger region of the fingers subdomain, a structurally conserved motif that forms part of the NTP entry tunnel and stabilizes interdomain architecture essential for viral RNA synthesis [119,120]. Mutations in this region are known to influence the electrostatic environment and conformational flexibility that guide nucleotide entry and analog recognition [119]. The R355Q substitution results in the loss of a positive charge, which may directly influence nucleotide positioning or the incorporation of UPGNUC255’s active triphosphate form, leading to resistance. Interestingly, R355 is a conserved residue among different flaviviruses, falls into the 20 amino acid conserved residues referred as “motif C” within bNLS of the N-terminal region of NS5 in Kunjin virus and was speculated to bind NS3 and other non-structural proteins such as NS2A. The deletion of this 20 amino acid sequence drastically reduced rescue of the defective virus [121,122]. Given its location in the polymerase domain, this mutation likely affects the nucleoside binding pocket, reducing UPGNUC255 selection while preserving essential polymerase function. Together, these findings strongly support that UPGNUC255 functions as a direct-acting nucleoside analog targeting the viral RdRp, and that R355 within a structurally sensitive region may represent a critical determinant of compound sensitivity and antiviral resistance. Importantly, mutations in this residue confer a fitness disadvantage making it unlikely to arise naturally. While the absence of *in vivo* data represents a limitation of the present study, *in vivo* validation constitutes an essential next step toward establishing the translational potential of these antiviral candidates. The current work focused on extensive *in vitro* screening, cross-cell-type validation, and mechanistic characterization. Ongoing efforts are directed at evaluating the pharmacokinetic profile, metabolic stability, and therapeutic efficacy of both the compounds in relevant animal models of flaviviral infection.

Altogether, this study identifies and characterizes novel nucleoside analogs with broad antiflaviviral activity, uncovers critical resistance mechanisms through NS5 mutations near and downstream of the GDD motif, and reveals a vulnerable region of the polymerase that modulates analog susceptibility while preserving replicative capacity. Notably, both compounds UPGNUC255 and UPGNUC558 exhibited consistent inhibition of viral RNA replication aligning well with their potent and broad antiviral activity observed across different DENV serotypes and cell models. Collectively, these findings not only advance the field of antiviral development but also underscore the need to understand resistance mechanisms and potential for cross resistance in the design of next-generation flavivirus therapeutics.

## Methods

### Cells

A549 obtained from ATCC (CCL-185) and Huh7.5 (C. Rice, Rockefeller) were cultured in DMEM, supplemented with 10% (v/v) fetal bovine serum, 1% (v/v) penicillin/streptomycin (Invitrogen), 1% (v/v) L-Glutamax (Invitrogen) and were maintained at 37°C and 5%. CO_2_. HepG2 cells (ATCC HB-8065) were obtained from ATCC and cultured in MEM, supplemented with 10% (v/v) fetal bovine serum, 1% (v/v) penicillin/streptomycin, 1% (v/v) L-glutamine, and were maintained at 37°C and 5% CO_2_. IMR90 cells (CCL-186) were obtained from ATCC and cultured in DMEM-20 (4.5g/L glucose with Na-pyruvate) supplemented with 20% (v/v) fetal bovine serum and were maintained at 37°C and 5% CO_2_. HEK293T cells were acquired from ATCC and propagated in DMEM supplemented with 10% heat-inactivated FBS and 1% (v/v) penicillin/streptomycin (Invitrogen), 1% (v/v) L-Glutamax (Invitrogen) and were maintained at 37°C and 5% CO_2_. Cell lines were validated to be free of Mycoplasma contamination using a Mycostrip kit (Invivogen).

Primary human keratinocytes and dermal fibroblasts were obtained from the UPenn Skin Biology and Diseases Resource-based Center (P30-AR069589). Briefly, neonatal foreskins were collected in DMEM, 20% FBS, antibiotic/antimycotic (Gibco) and gentamicin (Gibco). Foreskins were dissociated overnight in dispase (Stem Cell Technologies) to separate epidermis from dermis. Epidermal sheets were incubated in 0.05% trypsin-EDTA (Invitrogen), and dissociated keratinocytes were collected by centrifugation and resuspended in keratinocyte culture medium and Antibiotic/Antimycotic (Gibco). Keratinocyte culture medium contains a 1:1 mixture of Keratinocyte Serum Free Media supplemented with recombinant epidermal growth factor and bovine pituitary extract (Gibco 17005042) and Medium 154 (Gibco M154500) supplemented with human keratinocyte growth supplement (Gibco S0015) and maintained at 37°C and 5% CO_2_. Dermis was dissociated in collagenase followed by incubation in 0.5% trypsin-EDTA (Invitrogen). Dissociated fibroblasts were collected by centrifugation and resuspended in fibroblast culture medium containing DMEM, 10% FBS, 10 mM HEPES, supplemented with penicillin, streptomycin, and 250 µg/ml Fungizone (Gibco) and maintained at 37°C and 5% CO_2_. Keratinocytes and fibroblasts were used for experiments between passages 2–4.

### Virus stocks

DENV Type 2 (New Guinea C) from ATCC. DENV Type 1 Strain UIS 998 (NR-49713), DENV Type 3 Strain US/BID-V1043/2006 (NR-43282), DENV Type 4 Strain UIS 497 (NR-49724) and YFV 17D from BEI resources (ATCC). ZIKV (MR766) and Kunjin were obtained from Dr. R. Tesh (The World Reference Center for Emerging Viruses and Arboviruses (WRCEVA) at UTMB) and ZIKV (Puerto Rico) C. Coyne (Duke) and WNV (NY2000) M. Diamond (Washington University). All viruses were propagated in C636 and tittered by TCID_50_ assay performed on BHK-21 cells and sequence verified.

### Antibodies

Monoclonal antibodies against flavivirus glycoproteins (4G2) were generated UPenn in-house facility using hybridomas. Fluorescent secondary antibodies were from Life technologies. Hoechst 33342 was from Sigma-Aldrich.

### High-throughput screening

Cells were plated in 384 well plates 3,000 cells per well for Huh7.5, A549, HepG2 and IMR90. The next day, 50 μl of drugs were added to the final concentration of 50 μM. The library of nucleoside analogs were purchased from Granlen and Selleckchem. Positive controls NITD-008, AT-527 (10uM) and negative controls (DMSO) were spotted on each plate. One hour after the drug treatment, cells were infected with either DENV-2 (Huh7.5 MOI = 2, A549 MOI = 2, HepG2 MOI = 1 and IMR90 MOI = 5), WNV (Huh7.5 MOI = 5) for 24 h or with ZIKV (Huh7.5 MOI = 0.05) for 48h. Cells were fixed in 4% formaldehyde in PBS for 15 min at room temperature followed by 3x washes with PBS. Cells were blocked with 2% BSA in PBST for 60 minutes and incubated in primary antibody (anti-4G2) overnight at 4°C. Cells were washed 3x in PBST with an automated plate washer (BioTek) and incubated in secondary antibody (anti-mouse Alexa 488, 1:1,000 and Hoescht 33342) for 1 h at room temperature. Cells were washed 3x in PBST and imaged using an automated microscope (ImageXpress Micro, Molecular Devices) with a × 10 objective, and four sites per well were captured. The total number of cells and the number of infected cells were measured using the cell scoring module (MetaXpress 5.3.3), and the percentage of infected cells was calculated. The aggregated percent infection of the DMSO (*n* = 32) and 10 µM positive control wells (*n* = 16) on each assay plate were used to calculate *z*′-factors, as a measure of assay performance and data quality. Sample well infection was normalized to aggregated DMSO plate control wells and expressed as percentage of control (POC = (% infection_sample_/average % infection_DMSO_) × 100) and *Z* score (*Z* = (% Infection_sample_ − average % Infection_DMSO_)/Standard Deviation %infection_DMSO_) in Spotfire (PerkinElmer). Candidate hits were selected as compounds with >60% reduction in infection and maintained >80% cell viability in either replicate compared to DMSO control.

### Dose–response studies

Candidate drugs were repurchased as powders from Granlen, Selleckchem, MedChemExpress, and MedKoo and suspended in DMSO. Drugs were arrayed in 8-pt dose-response in 384 well plates. Infections were performed using the screening conditions. DMSO (n = 32) and 10 μM positive control drugs (n = 16) were included on each validation plate as controls for normalization. Infection at each drug concentration was normalized to aggregated DMSO plate control wells and expressed as percentage-of-control (POC = % Infection _sample_/Avg % Infection DMSO control). Cytotoxicity CC_50_ in the absence of viral infection was assessed using the ATPlite luminescence assay (PerkinElmer), which quantifies intracellular ATP levels as an indicator of metabolically active (viable) cells. Luminescence intensity, directly proportional to ATP content, served as a surrogate measure of cell viability.

A non-linear regression curve fit analysis (GraphPad Prism 9) was performed on the aggregated average POC Infection and cell viability from ≥ 2 independent experimental replicates versus the log_10_ transformed concentration values to calculate IC_50_ values for Infection and CC_50_ values for cell viability for each drug/cell line combination. Error bars represent the standard deviation of replicate data for each drug concentration tested in independent experiments. Selectivity index (SI) was calculated as a ratio of drug’s CC_50_ and IC_50_ values (SI = CC_50_/IC_50_).

### RT-qPCR

Huh7.5 (200,000 cells/well), A549 cells (200,000 cells/well), HepG2 (400,000 cells/well), IMR90 (200,000 cells/well), were plated in 12 well plates. Next day, cells were treated with indicated drugs to the final concentrations of 2 μM and 10 μM including positive controls in each experiment. One hour later cells were infected with DENV2 (Huh7.5 MOI = 0.05; A549 MOI = 0.05; HepG2 MOI = 0.5 and IMR90 MOI = 0.5). For Primary keratinocytes and fibroblasts 200,000 cells/well and 150,000 cells/well were plated in 12 well plates, respectively. 48 hours after plating cells were treated with indicated drugs to the final concentration of 10 μM including positive controls in each experiment. Cells were infected with DENV2 at MOI = 0.5 with spin at 1250 rcf for 1 hr. For other viruses, Huh7.5 cells were plated at 200,000 cells/well density and next day were infected with WNV (MOI = 0.5), ZIKA (MOI = 0.5), Kunjin (MOI = 0.5), YFV (MOI = 0.5), DENV1 (MOI = 1), DENV3 (MOI = 0.5), DENV4 (MOI = 0.5). Total RNA was purified using Trizol (Invitrogen) followed by RNA Clean and Concentrate kit (Zymo Researc) 24 hpi for Huh7.5, A549, HepG2 and IMR90 cells, whereas, 48 hpi for primary keratinocytes and fibroblasts cells. For cDNA synthesis, reverse transcription was performed with random hexamers and Moloney murine leukemia virus (M-MLV) reverse transcriptase (Invitrogen). Gene specific primers to each virus (listed in S3 Table) and SYBR green master mix (Applied Biosystems) were used to amplify viral RNA and 18S rRNA primers were used to amplify cellular RNA in the QuantStudio 6 Flex RT-PCR system (Applied Biosystems). Relative quantities of viral and cellular RNA were calculated using the standard curve method (Larionov et al., 2005). Viral RNA was normalized to human 18S RNA for each sample.

### Selection of resistance mutants

KUNV was sequentially passaged in Huh7.5 cells in gradually increasing concentration of nucleoside analogs to select for drug resistance. In brief, Huh7.5 cells were plated at 100,000 cells/well monolayer in 24 well plates. 24h later the cells are infected with KUNV in media for 2h at 37°C and washed with cold PBS and media replenished with vehicle or indicated nucleoside analog. 48 hours later the supernatants were harvested from each well after the occurrence of cytopathic effect (CPE) from day 2 onwards. 100 µl of supernatant is used to reinfect fresh cell monolayers for subsequent passages, gradually increasing drug concentration up to 10 times the IC_50_ value. Virus was expanded in C6/36 cells and titered.

### Whole Genome Sequencing data analysis

Whole-genome sequence analysis was performed on virus stocks, including mutants and DMSO control virus strains. Short read single-end viral whole genome sequencing was performed on a NextSeq 2000 P2 100 Cycle kit with a read length of 118 bp. Fastq files were generated and then compared to a reference sequence for KUNV (AY274504). Reads were then mapped to the reference genome by breseq 0.36.0 (generated by the Barrick lab) using bowtie2 2.4.1, and subsequent identification of mutations relative to the reference sequence were done using breseq 0.36.0 in polymorphism mode (assuming mixed sample populations of variants sequenced) with the number of processors to use in multithreaded steps set to 8 and a 5% mutation frequency cutoff. The breseq gdtools *compare* command was then used to generate html files reporting mutation frequency across samples relative to the reference genome [123].

### Plasmids and generation of mutants

WNV lineage II Replicon encoding GFP was a generous gift from Dr. Ted Pierson at the NIH. We used wild type and S135A (protease dead) mutant of NS3 and the D668A (polymerase dead) mutant of NS5 were used as replication-deficient controls, as these mutations render the virus incapable of RNA replication. To generate mutants, primers were ordered from Integrated DNA Technologies (IDT) encoding R → 355Q, A → 534S or S → 604T mutations in NS5. Site-directed mutagenesis was performed using InFusion cloning (Takara). The sequence of all replicon plasmids was validated using Oxford nanopore sequencing (Plasmidsaurus).

### Cell transfection and replicon assays

HEK293T cells were seeded into 6-well plates and transfected with replicon plasmid using Lipofectamine 3000 reagent and treated with the indicated nucleoside analogs and vehicle (DMSO). 24 h post-transfection, cells were replated in 96-well plates with the addition of indicated nucleoside analogs for automated immunofluorescence analysis. 48 hours post-transfection cells were fixed with 4% paraformaldehyde and processed for automated imaging in an automated microscope (ImageXpress Micro, Molecular Devices). The resulting images were analyzed using MetaXpress software.

### Modeling

Molecular modeling of the Kunjin structure was based on an AlphaFold3 model of Dengue2 (WLD15668.1). We used the DENV2 sequence because the KUNV sequence was rejected by the biosecurity sequence filters and could not be directly modeled. Nevertheless, the Dengue polymerase model (66% identity to Kunjin), with included RNA strands and incoming ATP nucleotide, provided a template for modeling of the Kunjin enzyme. Homology modeling was performed in the MOE environment (version 2022, Chemical Computing Group). Locations of mutations and active site residues were visualized on the homology model.

### Quantification and statistical analysis

P-values for RT–qPCR experiments were obtained by performing one-way ANOVA, assuming equal SD, with multiple comparisons using Dunnett’s correction for multiple comparisons on log_10_-transformed values on relative copy number values from at least three independent experiments. For TCID50 experiments, statistical significance was determined using one-way ANOVA with Dunnett’s correction for multiple comparisons was performed on titers from at least three independent experiments. For multiple comparisons, each condition was compared with vehicle control (DMSO). Visualization of data was performed using GraphPad Prism 9. The statistical parameters for experiments are described in the figure legends. n indicates the number of independent experiments performed, and significance is defined as P < 0.05.

## Supporting information

S1 Fig Dose response analysis of control drugs. **A.** Chemical structures of control nucleoside analogs MK-0608, NITD008 and AT-527 **B.** Dose response analysis of MK0608, NITD008 and AT-527 in Huh7.5, HepG2, A549, and IMR90 cells infected with DENV2 at MOI = 2 (Huh7.5 and A549), MOI = 1 (HepG2), and MOI = 5 (IMR90). At 24 hours post-infection (hpi), viral antigen (4G2) and quantified via automated fluorescence microscopy and plotted as POC infection (black) and cell viability (green). **C.** Table of IC_50_, CC_50_, and SI values for control drugs tested in Huh7.5, HepG2, A549, and IMR90 cells infected with DENV2 as in B. SI shown for CC_50_ (ATPlite)/IC_50_.
(TIF)

S2 Fig Chemical structures and dose response validation of indicated nucleoside analogs. **A.** Chemical structures of 16 drugs selected from the primary screen having SI > 10 in at least one cell model (excluding Ribavirin, UPGNUC396). **B.** Dose response analysis of selected candidates in Huh7.5, HepG2, A549, and IMR90 cells against DENV2 infection. POC percent infection (black) POC cell viability (green).
(TIF)

S3 Fig Orthogonal validation of nucleoside analogs activity across various cell models: Indicated cells were pretreated with the specified compounds. **A-D.**10 μM or **E**. 2 μM or DMSO vehicle control infected with DENV2 infection in Huh7.5 (MOI = 0.05), HepG2 (MOI = 0.5), A549 (MOI = 0.05), and IMR90 (MOI = 0.5) for 24 hpi, and subject to RT-qPCR. Data are presented as mean ± SD, showing viral RNA levels relative to the vehicle control (n ≥ 1–3 independent biological replicates). Statistical significance was determined on n ≥ 3 by one-way ANOVA with Dunnett’s correction for multiple comparisons on log_10_-transformed values (*P < 0.05, **P < 0.01, ***P < 0.001, ****P < 0.0001).
(TIF)

S4 Fig Orthogonal validation of nucleoside analog activity across DENV serotypes and other flaviviruses: qRT-PCR analysis of dengue serotypes. **A.** DENV1, **B.** DENV3 and **C.** DENV4 infection (MOI = 0.5 each, 24 hpi) in Huh7.5 cells pretreated with the indicated compounds (10 μM) or DMSO vehicle control. Data are presented as mean ± SD, showing viral RNA levels relative to the vehicle control (n ≥ 3 independent biological replicates). Statistical significance was determined for n ≥ 3 by one-way ANOVA with Dunnett’s correction for multiple comparisons on log_10_-transformed values (*P < 0.05, **P < 0.01, ***P < 0.001, ****P < 0.0001). qRT-PCR analysis of **D.** WNV infection **E.** KUNV infection **F.** ZIKA (Puerto Rico) infection **G.** ZIKA (MR766) infection and **H.** YFV (17D) infection at MOI = 0.5 each in Huh7.5 cells pretreated with the indicated compounds (10 μM) or DMSO vehicle control at 24 hpi. qPCR data are presented as mean ± SD, showing viral RNA levels relative to the vehicle control (n ≥ 1–3 independent biological replicates). Statistical significance was determined for n ≥ 3 by one-way ANOVA with Dunnett’s correction for multiple comparisons on log_10_-transformed values (*P < 0.05, **P < 0.01, ***P < 0.001, ****P < 0.0001).
(TIF)

S5 Fig Nucleoside analog activity in primary cells. **A.** RT-qPCR analysis in primary keratinocytes pretreated with the indicated compounds (10 μM) or DMSO vehicle control were infected with DENV2 (MOI = 0.5) and subject to RT-qPCR 48hpi. **B.** Primary fibroblasts pretreated with the indicated compounds (10 μM) or DMSO vehicle control were infected with DENV2 (MOI = 0.5) and subject to RT-qPCR 48hpi. **C.** Primary keratinocytes pretreated with the indicated compounds (10 μM) or DMSO vehicle control were infected with WNV (MOI = 0.5) and subject to RT-qPCR 48hpi. For all qPCR, data are presented as mean ± SD, showing viral RNA levels relative to the vehicle control (n ≥ 1–3 independent biological replicates). Statistical significance was determined for n ≥ 3 by one-way ANOVA with Dunnett’s correction for multiple comparisons on log_10_transformed values (*P < 0.05, **P < 0.01, ***P < 0.001, ****P < 0.0001).
(TIF)

S6 Fig Chemical structures of 2’ substituted nucleoside analogs. (TIF)

S7 Fig Chemical structures of additional nucleoside analogs. (TIF)

S8 Fig WNV and ZIKV infect A549 and IMR90 cells. Representative microscopy images for WNV (NY2000) and ZIKA (MR766) infection in A549 and IMR90 cells. Cells were treated with either DMSO or NITD008 (10uM) and fixed 24 hpi and stained for viral infection (4G2, green) and cell number (Hoechst 33342, blue). 10 × magnification.
(TIF)

S9 Fig Dose response analysis of UPGNUC255 derivatives against flaviviruses. **A.** Chemical structures. **B.** Dose response analysis of indicated nucleoside analogs in Huh7.5 cells infected with DENV2 or KUNV for 24h and subject to automated microscopy or at 48h processed for cell viability (ATPlite). POC percent infection (black) POC cell viability (green). **C.** Table of IC_50_, CC_50_, and SI values for indicated drugs tested in Huh7.5 cells infected with DENV2 and KUNV. SI shown for CC_50_ (ATPlite)/IC_50_.
(TIF)

S10 Fig Conserved residues are involved in nucleoside analog selection. **A-C.** Alignment of sequences in the NS5 region of KUNV with indicated flaviviruses: Kunjin (AAP78942.1), West Nile (YP_001527887.1), DENV1 (QMW69322.1), DENV2 NGC (NP_739590.2), DENV3 (QUD37256.1), DENV4 (UCQ65264.1), Japanese encephalitis virus (JEV) (AIN36651.1), ZIKA Virus (AMQ34004.1). Conserved amino acids are highlighted in yellow and three amino acids **A.** R355, **B.** A534 and **C.** S604 are indicated in the red box. **D.** Traces of sanger sequencing are showing Kunjin NS5 RdRp gene mutation in UPGUC255_R3 and UPGNUC255_R6 resistant strains where Arginine-355 residue has been substituted by Glutamine (dark orange), *represented the substituted A in place of G. **E.** HEK293T cells were pretreated with the indicated nucleosides or vehicle were infected with KUNV (MOI = 0.5), and 24 hpi subject to RT-qPCR. Data are presented as mean ± SD, showing viral RNA levels relative to the vehicle control (n = 2).
(TIF)

S1 Table Primary screening data. (XLSX)

S2 Table Antiviral testing across flaviviruses. (XLSX)

S3 Table Primers used in this study. (XLSX)

S4 Table Raw data for manuscript. (XLSX)

## References

[1] Cattarino L, Rodriguez-Barraquer I, Imai N, Cummings DAT, Ferguson NM. Mapping global variation in dengue transmission intensity. Sci Transl Med. 2020;12(528):eaax4144. doi: 10.1126/scitranslmed.aax4144 31996463

[2] Paz-Bailey G, Adams LE, Deen J, Anderson KB, Katzelnick LC. Dengue. Lancet. 2024;403(10427):667–82. doi: 10.1016/S0140-6736(23)02576-X 38280388 PMC12372472

[3] Stanaway JD, Shepard DS, Undurraga EA, Halasa YA, Coffeng LE, Brady OJ, et al. The global burden of dengue: an analysis from the Global Burden of Disease Study 2013. Lancet Infect Dis. 2016;16(6):712–23. doi: 10.1016/S1473-3099(16)00026-8 26874619 PMC5012511

[4] Yang X, Quam MBM, Zhang T, Sang S. Global burden for dengue and the evolving pattern in the past 30 years. J Travel Med. 2021;28:1–11. doi: 10.1093/jtm/taab146 34510205

[5] eClinicalMedicine. Dengue as a growing global health concern. EClinicalMedicine. 2024;77:102975. doi: 10.1016/j.eclinm.2024.102975 39649133 PMC11625016

[6] Rocklöv J, Tozan Y. Climate change and the rising infectiousness of dengue. Emerg Top Life Sci. 2019;3(2):133–42. doi: 10.1042/ETLS20180123 33523146 PMC7288996

[7] Brady OJ, Hay SI. The Global Expansion of Dengue: How Aedes aegypti Mosquitoes Enabled the First Pandemic Arbovirus. Annu Rev Entomol. 2020;65:191–208. doi: 10.1146/annurev-ento-011019-024918 31594415

[8] Bhatt S, Gething PW, Brady OJ, Messina JP, Farlow AW, Moyes CL, et al. The global distribution and burden of dengue. Nature. 2013;496(7446):504–7. doi: 10.1038/nature12060 23563266 PMC3651993

[9] Kaye AR, Obolski U, Sun L, Hart WS, Hurrell JW, Tildesley MJ, et al. The impact of natural climate variability on the global distribution of Aedes aegypti: a mathematical modelling study. Lancet Planet Health. 2024;8(12):e1079–87. doi: 10.1016/S2542-5196(24)00238-9 39674197 PMC7617884

[10] Simmons CP, Farrar JJ, Nguyen van VC, Wills B. Dengue. N Engl J Med. 2012;366(15):1423–32. doi: 10.1056/NEJMra1110265 22494122

[11] Guzman MG, Harris E. Dengue. Lancet. 2015;385(9966):453–65. doi: 10.1016/S0140-6736(14)60572-9 25230594

[12] Clapham HE, Cummings DAT, Johansson MA. Immune status alters the probability of apparent illness due to dengue virus infection: Evidence from a pooled analysis across multiple cohort and cluster studies. PLoS Negl Trop Dis. 2017;11(9):e0005926. doi: 10.1371/journal.pntd.0005926 28953902 PMC5633199

[13] Nisalak A, Clapham HE, Kalayanarooj S, Klungthong C, Thaisomboonsuk B, Fernandez S, et al. Forty Years of Dengue Surveillance at a Tertiary Pediatric Hospital in Bangkok, Thailand, 1973-2012. Am J Trop Med Hyg. 2016;94(6):1342–7. doi: 10.4269/ajtmh.15-0337 27022151 PMC4889755

[14] Soo K-M, Khalid B, Ching S-M, Chee H-Y. Meta-Analysis of Dengue Severity during Infection by Different Dengue Virus Serotypes in Primary and Secondary Infections. PLoS One. 2016;11(5):e0154760. doi: 10.1371/journal.pone.0154760 27213782 PMC4877104

[15] Tricou V, Yu D, Reynales H, Biswal S, Saez-Llorens X, Sirivichayakul C, et al. Long-term efficacy and safety of a tetravalent dengue vaccine (TAK-003): 4·5-year results from a phase 3, randomised, double-blind, placebo-controlled trial. Lancet Glob Health. 2024;12(2):e257–70. doi: 10.1016/S2214-109X(23)00522-3 38245116

[16] Lindenbach BD, Rice CM. Molecular biology of flaviviruses. Adv Virus Res. 2003;59:23–61. doi: 10.1016/s0065-3527(03)59002-9 14696326

[17] Pierson TC, Diamond MS. The continued threat of emerging flaviviruses. Nat Microbiol. 2020;5(6):796–812. doi: 10.1038/s41564-020-0714-0 32367055 PMC7696730

[18] Wu Z, He Y, Wang T, Wang M, Cheng A, Chen S. DENV and ZIKV infection: Species specificity and broad cell tropism. Virology. 2024;600:110276. doi: 10.1016/j.virol.2024.110276 39467358

[19] Mlakar J, Korva M, Tul N, Popović M, Poljšak-Prijatelj M, Mraz J, et al. Zika Virus Associated with Microcephaly. N Engl J Med. 2016;374(10):951–8. doi: 10.1056/NEJMoa1600651 26862926

[20] Krauer F, Riesen M, Reveiz L, Oladapo OT, Martínez-Vega R, Porgo TV, et al. Zika Virus Infection as a Cause of Congenital Brain Abnormalities and Guillain-Barré Syndrome: Systematic Review. PLoS Med. 2017;14(1):e1002203. doi: 10.1371/journal.pmed.1002203 28045901 PMC5207634

[21] Guo Z, Jing W, Liu J, Liu M. The global trends and regional differences in incidence of Zika virus infection and implications for Zika virus infection prevention. PLoS Negl Trop Dis. 2022;16(10):e0010812. doi: 10.1371/journal.pntd.0010812 36269778 PMC9586358

[22] Bai F, Thompson EA, Vig PJS, Leis AA. Current Understanding of West Nile Virus Clinical Manifestations, Immune Responses, Neuroinvasion, and Immunotherapeutic Implications. Pathogens. 2019;8(4):193. doi: 10.3390/pathogens8040193 31623175 PMC6963678

[23] Pesko KN, Ebel GD. West Nile virus population genetics and evolution. Infect Genet Evol. 2012;12(2):181–90. doi: 10.1016/j.meegid.2011.11.014 22226703 PMC4494105

[24] Gray TJ, Burrow JN, Markey PG, Whelan PI, Jackson J, Smith DW, et al. West nile virus (Kunjin subtype) disease in the northern territory of Australia--a case of encephalitis and review of all reported cases. Am J Trop Med Hyg. 2011;85(5):952–6. doi: 10.4269/ajtmh.2011.11-0165 22049056 PMC3205648

[25] Srivastava KS, Jeswani V, Pal N, Bohra B, Vishwakarma V, Bapat AA, et al. Japanese Encephalitis Virus: An Update on the Potential Antivirals and Vaccines. Vaccines (Basel). 2023;11(4):742. doi: 10.3390/vaccines11040742 37112654 PMC10146181

[26] Zhu Y, He Z, Qi Z. Virus-host Interactions in Early Japanese Encephalitis Virus Infection. Virus Res. 2023;331:199120. doi: 10.1016/j.virusres.2023.199120 37086856 PMC10345573

[27] Kleinert RDV, Montoya-Diaz E, Khera T, Welsch K, Tegtmeyer B, Hoehl S, et al. Yellow Fever: Integrating Current Knowledge with Technological Innovations to Identify Strategies for Controlling a Re-Emerging Virus. Viruses. 2019;11(10):960. doi: 10.3390/v11100960 31627415 PMC6832525

[28] Garske T, Van Kerkhove MD, Yactayo S, Ronveaux O, Lewis RF, Staples JE, et al. Yellow Fever in Africa: estimating the burden of disease and impact of mass vaccination from outbreak and serological data. PLoS Med. 2014;11(5):e1001638. doi: 10.1371/journal.pmed.1001638 24800812 PMC4011853

[29] Surasombatpattana P, Hamel R, Patramool S, Luplertlop N, Thomas F, Desprès P, et al. Dengue virus replication in infected human keratinocytes leads to activation of antiviral innate immune responses. Infect Genet Evol. 2011;11(7):1664–73. doi: 10.1016/j.meegid.2011.06.009 21722754

[30] Duangkhae P, Erdos G, Ryman KD, Watkins SC, Falo LD Jr, Marques ETA Jr, et al. Interplay between Keratinocytes and Myeloid Cells Drives Dengue Virus Spread in Human Skin. J Invest Dermatol. 2018;138(3):618–26. doi: 10.1016/j.jid.2017.10.018 29106931

[31] Helgers LC, Keijzer NCH, van Hamme JL, Sprokholt JK, Geijtenbeek TBH. Dengue Virus Infects Human Skin Langerhans Cells through Langerin for Dissemination to Dendritic Cells. J Invest Dermatol. 2024;144(5):1099-1111.e3. doi: 10.1016/j.jid.2023.09.287 37979773

[32] Begum F, Das S, Mukherjee D, Mal S, Ray U. Insight into the Tropism of Dengue Virus in Humans. Viruses. 2019;11(12):1136. doi: 10.3390/v11121136 31835302 PMC6950149

[33] Hodge K, Tunghirun C, Kamkaew M, Limjindaporn T, Yenchitsomanus P-T, Chimnaronk S. Identification of a Conserved RNA-dependent RNA Polymerase (RdRp)-RNA Interface Required for Flaviviral Replication. J Biol Chem. 2016;291(33):17437–49. doi: 10.1074/jbc.M116.724013 27334920 PMC5016140

[34] Eyer L, Nencka R, de Clercq E, Seley-Radtke K, Růžek D. Nucleoside analogs as a rich source of antiviral agents active against arthropod-borne flaviviruses. Antivir Chem Chemother. 2018;26. doi: 10.1177/2040206618761299 29534608 PMC5890575

[35] Carroll SS, Olsen DB. Nucleoside analog inhibitors of hepatitis C virus replication. Infect Disord Drug Targets. 2006;6(1):17–29. doi: 10.2174/187152606776056698 16787301

[36] Carroll SS, Tomassini JE, Bosserman M, Getty K, Stahlhut MW, Eldrup AB, et al. Inhibition of hepatitis C virus RNA replication by 2’-modified nucleoside analogs. J Biol Chem. 2003;278(14):11979–84. doi: 10.1074/jbc.M210914200 12554735

[37] Klumpp K, Lévêque V, Le Pogam S, Ma H, Jiang W-R, Kang H, et al. The novel nucleoside analog R1479 (4’-azidocytidine) is a potent inhibitor of NS5B-dependent RNA synthesis and hepatitis C virus replication in cell culture. J Biol Chem. 2006;281(7):3793–9. doi: 10.1074/jbc.M510195200 16316989

[38] Kabinger F, Stiller C, Schmitzová J, Dienemann C, Kokic G, Hillen HS, et al. Mechanism of molnupiravir-induced SARS-CoV-2 mutagenesis. Nat Struct Mol Biol. 2021;28(9):740–6. doi: 10.1038/s41594-021-00651-0 34381216 PMC8437801

[39] Yin W, Mao C, Luan X, Shen D-D, Shen Q, Su H, et al. Structural basis for inhibition of the RNA-dependent RNA polymerase from SARS-CoV-2 by remdesivir. Science. 2020;368(6498):1499–504. doi: 10.1126/science.abc1560 32358203 PMC7199908

[40] Gordon CJ, Tchesnokov EP, Woolner E, Perry JK, Feng JY, Porter DP, et al. Remdesivir is a direct-acting antiviral that inhibits RNA-dependent RNA polymerase from severe acute respiratory syndrome coronavirus 2 with high potency. J Biol Chem. 2020;295(20):6785–97. doi: 10.1074/jbc.RA120.013679 32284326 PMC7242698

[41] Saiz J-C, Oya NJ de, Blázquez A-B, Escribano-Romero E, Martín-Acebes MA. Host-Directed Antivirals: A Realistic Alternative to Fight Zika Virus. Viruses. 2018;10(9):453. doi: 10.3390/v10090453 30149598 PMC6163279

[42] Ji X, Li Z. Medicinal chemistry strategies toward host targeting antiviral agents. Med Res Rev. 2020;40(5):1519–57. doi: 10.1002/med.21664 32060956 PMC7228277

[43] Geraghty RJ, Aliota MT, Bonnac LF. Broad-Spectrum Antiviral Strategies and Nucleoside Analogues. Viruses. 2021;13(4):667. doi: 10.3390/v13040667 33924302 PMC8069527

[44] Roberts SK, Cooksley G, Dore GJ, Robson R, Shaw D, Berns H, et al. Robust antiviral activity of R1626, a novel nucleoside analog: a randomized, placebo-controlled study in patients with chronic hepatitis C. Hepatology. 2008;48(2):398–406. doi: 10.1002/hep.22321 18553458

[45] Chen Y-L, Abdul Ghafar N, Karuna R, Fu Y, Lim SP, Schul W, et al. Activation of peripheral blood mononuclear cells by dengue virus infection depotentiates balapiravir. J Virol. 2014;88(3):1740–7. doi: 10.1128/JVI.02841-13 24257621 PMC3911617

[46] Nguyen NM, Tran CNB, Phung LK, Duong KTH, Huynh H le A, Farrar J, et al. A randomized, double-blind placebo controlled trial of balapiravir, a polymerase inhibitor, in adult dengue patients. J Infect Dis. 2013;207(9):1442–50. doi: 10.1093/infdis/jis470 22807519 PMC3610419

[47] Qing J, Luo R, Wang Y, Nong J, Wu M, Shao Y, et al. Resistance analysis and characterization of NITD008 as an adenosine analog inhibitor against hepatitis C virus. Antiviral Res. 2016;126:43–54. doi: 10.1016/j.antiviral.2015.12.010 26724382

[48] Yin Z, Chen Y-L, Schul W, Wang Q-Y, Gu F, Duraiswamy J, et al. An adenosine nucleoside inhibitor of dengue virus. Proc Natl Acad Sci U S A. 2009;106(48):20435–9. doi: 10.1073/pnas.0907010106 19918064 PMC2787148

[49] Barrows NJ, Campos RK, Powell ST, Prasanth KR, Schott-Lerner G, Soto-Acosta R, et al. A Screen of FDA-Approved Drugs for Inhibitors of Zika Virus Infection. Cell Host Microbe. 2016;20(2):259–70. doi: 10.1016/j.chom.2016.07.004 27476412 PMC4993926

[50] Lo MK, Shi P-Y, Chen Y-L, Flint M, Spiropoulou CF. In vitro antiviral activity of adenosine analog NITD008 against tick-borne flaviviruses. Antiviral Res. 2016;130: 46–9. doi: 10.1016/j.antiviral.2016.03.013 27016316 PMC5096641

[51] Deng Y-Q, Zhang N-N, Li C-F, Tian M, Hao J-N, Xie X-P, et al. Adenosine Analog NITD008 Is a Potent Inhibitor of Zika Virus. Open Forum Infect Dis. 2016;3(4):ofw175. doi: 10.1093/ofid/ofw175 27747251 PMC5063548

[52] Lissane Eddine FZ, Mathez G, Carlen V, Dolci I, Sachetto Fernandes R, Schutzer Godoy A, et al. Identification of pan-flavivirus compounds from drug repurposing. Antiviral Res. 2025;240:106205. doi: 10.1016/j.antiviral.2025.106205 40451519

[53] Schul W, Liu W, Xu H-Y, Flamand M, Vasudevan SG. A dengue fever viremia model in mice shows reduction in viral replication and suppression of the inflammatory response after treatment with antiviral drugs. J Infect Dis. 2007;195(5):665–74. doi: 10.1086/511310 17262707

[54] Mateo R, Nagamine CM, Kirkegaard K. Suppression of Drug Resistance in Dengue Virus. mBio. 2015;6(6):e01960–15. doi: 10.1128/mBio.01960-15 26670386 PMC4701834

[55] Carroll SS, Ludmerer S, Handt L, Koeplinger K, Zhang NR, Graham D, et al. Robust antiviral efficacy upon administration of a nucleoside analog to hepatitis C virus-infected chimpanzees. Antimicrob Agents Chemother. 2009;53(3):926–34. doi: 10.1128/AAC.01032-08 19075052 PMC2650549

[56] Eyer L, Valdés JJ, Gil VA, Nencka R, Hřebabecký H, Šála M, et al. Nucleoside inhibitors of tick-borne encephalitis virus. Antimicrob Agents Chemother. 2015;59(9):5483–93. doi: 10.1128/AAC.00807-15 26124166 PMC4538560

[57] Wu R, Smidansky ED, Oh HS, Takhampunya R, Padmanabhan R, Cameron CE, et al. Synthesis of a 6-methyl-7-deaza analogue of adenosine that potently inhibits replication of polio and dengue viruses. J Med Chem. 2010;53(22):7958–66. doi: 10.1021/jm100593s 20964406 PMC2990348

[58] Zhou X-J, Lickliter J, Montrond M, Ishak L, Pietropaolo K, James D, et al. First-in-human trial evaluating safety and pharmacokinetics of AT-752, a novel nucleotide prodrug with pan-serotype activity against dengue virus. Antimicrob Agents Chemother. 2024;68(5):e0161523. doi: 10.1128/aac.01615-23 38526047 PMC11064583

[59] Good SS, Shannon A, Lin K, Moussa A, Julander JG, La Colla P, et al. Evaluation of AT-752, a Double Prodrug of a Guanosine Nucleotide Analog with In Vitro and In Vivo Activity against Dengue and Other Flaviviruses. Antimicrob Agents Chemother. 2021;65(11):e0098821. doi: 10.1128/AAC.00988-21 34424050 PMC8522752

[60] Chazot A, Zimberger C, Feracci M, Moussa A, Good S, Sommadossi J-P, et al. The activation cascade of the broad-spectrum antiviral bemnifosbuvir characterized at atomic resolution. PLoS Biol. 2024;22(8):e3002743. doi: 10.1371/journal.pbio.3002743 39190717 PMC11349198

[61] Toussi SS, Hammond JL, Gerstenberger BS, Anderson AS. Therapeutics for COVID-19. Nat Microbiol. 2023;8(5):771–86. doi: 10.1038/s41564-023-01356-4 37142688

[62] Horga A, Saenz R, Yilmaz G, Simón-Campos A, Pietropaolo K, Stubbings WJ, et al. Oral bemnifosbuvir (AT-527) vs placebo in patients with mild-to-moderate COVID-19 in an outpatient setting (MORNINGSKY). Future Virol. 2023;:10.2217/fvl-2023–0115. doi: 10.2217/fvl-2023-0115 37928891 PMC10621114

[63] Stein DS, Moore KH. Phosphorylation of nucleoside analog antiretrovirals: a review for clinicians. Pharmacotherapy. 2001;21(1):11–34. doi: 10.1592/phco.21.1.11.34439 11191730

[64] Suzuki S, Marumo S. Chemical Structure of Tubercidin. J Antibiot Ser A. 1960;13:360. doi: 10.11554/antibioticsa.13.5_360

[65] Bergstrom DE, Brattesani AJ, Ogawa MK, Reddy PA, Schweickert MJ, Balzarini J, et al. Antiviral activity of C-5 substituted tubercidin analogues. J Med Chem. 1984;27(3):285–92. doi: 10.1021/jm00369a010 6699874

[66] Eyer L, Kondo H, Zouharova D, Hirano M, Valdés JJ, Muto M, et al. Escape of Tick-Borne Flavivirus from 2’-C-Methylated Nucleoside Antivirals Is Mediated by a Single Conservative Mutation in NS5 That Has a Dramatic Effect on Viral Fitness. J Virol. 2017;91(21):e01028–17. doi: 10.1128/JVI.01028-17 28814513 PMC5640847

[67] Noisakran S, Onlamoon N, Songprakhon P, Hsiao H-M, Chokephaibulkit K, Perng GC. Cells in dengue virus infection in vivo. Adv Virol. 2010;2010:164878. doi: 10.1155/2010/164878 22331984 PMC3276057

[68] Aye KS, Charngkaew K, Win N, Wai KZ, Moe K, Punyadee N, et al. Pathologic highlights of dengue hemorrhagic fever in 13 autopsy cases from Myanmar. Hum Pathol. 2014;45(6):1221–33. doi: 10.1016/j.humpath.2014.01.022 24767772

[69] Balsitis SJ, Coloma J, Castro G, Alava A, Flores D, McKerrow JH, et al. Tropism of dengue virus in mice and humans defined by viral nonstructural protein 3-specific immunostaining. Am J Trop Med Hyg. 2009;80(3):416–24. doi: 10.4269/ajtmh.2009.80.416 19270292

[70] Jessie K, Fong MY, Devi S, Lam SK, Wong KT. Localization of dengue virus in naturally infected human tissues, by immunohistochemistry and in situ hybridization. J Infect Dis. 2004;189(8):1411–8. doi: 10.1086/383043 15073678

[71] Tongluan N, Ramphan S, Wintachai P, Jaresitthikunchai J, Khongwichit S, Wikan N, et al. Involvement of fatty acid synthase in dengue virus infection. Virol J. 2017;14(1):28. doi: 10.1186/s12985-017-0685-9 28193229 PMC5307738

[72] Meertens L, Carnec X, Lecoin MP, Ramdasi R, Guivel-Benhassine F, Lew E, et al. The TIM and TAM families of phosphatidylserine receptors mediate dengue virus entry. Cell Host Microbe. 2012;12(4):544–57. doi: 10.1016/j.chom.2012.08.009 23084921 PMC3572209

[73] Eyer L, Šmídková M, Nencka R, Neča J, Kastl T, Palus M, et al. Structure-activity relationships of nucleoside analogues for inhibition of tick-borne encephalitis virus. Antiviral Res. 2016;133:119–29. doi: 10.1016/j.antiviral.2016.07.018 27476046

[74] Good SS, Moussa A, Zhou X-J, Pietropaolo K, Sommadossi J-P. Preclinical evaluation of AT-527, a novel guanosine nucleotide prodrug with potent, pan-genotypic activity against hepatitis C virus. PLoS ONE. 2020;15(1):e0227104. doi: 10.1371/journal.pone.0227104 PMC6949113 31914458

[75] Leyssen P, Balzarini J, De Clercq E, Neyts J. The predominant mechanism by which ribavirin exerts its antiviral activity in vitro against flaviviruses and paramyxoviruses is mediated by inhibition of IMP dehydrogenase. J Virol. 2005;79(3):1943–7. doi: 10.1128/JVI.79.3.1943-1947.2005 15650220 PMC544097

[76] Crance JM, Scaramozzino N, Jouan A, Garin D. Interferon, ribavirin, 6-azauridine and glycyrrhizin: antiviral compounds active against pathogenic flaviviruses. Antiviral Res. 2003;58(1):73–9. doi: 10.1016/s0166-3542(02)00185-7 12719009

[77] Lee HW, Tchesnokov EP, Stevens LJ, Hughes TM, Diefenbacher MV, Woolner E, et al. Mechanism and spectrum of inhibition of viral polymerases by 2’-deoxy-2’-β-fluoro-4’-azidocytidine or azvudine. NAR Mol Med. 2025;2(3):ugaf029. doi: 10.1093/narmme/ugaf029 40900943 PMC12400934

[78] Wang Q, Liu X, Wang Q, Zhang Y, Jiang J, Guo X, et al. FNC, a novel nucleoside analogue inhibits cell proliferation and tumor growth in a variety of human cancer cells. Biochem Pharmacol. 2011;81(7):848–55. doi: 10.1016/j.bcp.2011.01.001 21219886

[79] Meng Y, Sun N, Liang L, Yu B, Chang J. 2’-Fluorinated nucleoside chemistry for new drug discovery: achievements and prospects. Natl Sci Rev. 2024;11(10):nwae331. doi: 10.1093/nsr/nwae331 39526027 PMC11546638

[80] Zhang J-L, Li Y-H, Wang L-L, Liu H-Q, Lu S-Y, Liu Y, et al. Azvudine is a thymus-homing anti-SARS-CoV-2 drug effective in treating COVID-19 patients. Signal Transduct Target Ther. 2021;6(1):414. doi: 10.1038/s41392-021-00835-6 34873151 PMC8646019

[81] Toschi L, Finocchiaro G, Bartolini S, Gioia V, Cappuzzo F. Role of gemcitabine in cancer therapy. Future Oncol. 2005;1(1):7–17. doi: 10.1517/14796694.1.1.7 16555971

[82] De Clercq E, Bernaerts R, Bergstrom DE, Robins MJ, Montgomery JA, Holy A. Antirhinovirus activity of purine nucleoside analogs. Antimicrob Agents Chemother. 1986;29(3):482–7. doi: 10.1128/AAC.29.3.482 3013084 PMC180418

[83] Wu F, Cheng W, Zhao F, Tang M, Diao Y, Xu R. Association of N6-methyladenosine with viruses and related diseases. Virol J. 2019;16(1):133. doi: 10.1186/s12985-019-1236-3 31711514 PMC6849232

[84] Dang W, Xie Y, Cao P, Xin S, Wang J, Li S, et al. N6-Methyladenosine and Viral Infection. Front Microbiol. 2019;10:417. doi: 10.3389/fmicb.2019.00417 30891023 PMC6413633

[85] Nair V, Shu Q. Inosine monophosphate dehydrogenase as a probe in antiviral drug discovery. Antivir Chem Chemother. 2007;18(5):245–58. doi: 10.1177/095632020701800501 18046958

[86] Schwarz S, Siewert B, Csuk R, Rauter AP. New antitumor 6-chloropurine nucleosides inducing apoptosis and G2/M cell cycle arrest. Eur J Med Chem. 2015;90:595–602. doi: 10.1016/j.ejmech.2014.11.019 25499928

[87] Eyer L, Svoboda P, Balvan J, Vičar T, Raudenská M, Štefánik M, et al. Broad-Spectrum Antiviral Activity of 3’-Deoxy-3’-Fluoroadenosine against Emerging Flaviviruses. Antimicrob Agents Chemother. 2021;65(2):e01522–20. doi: 10.1128/AAC.01522-20 33229424 PMC7848998

[88] Holmes EC, Burch SS. The causes and consequences of genetic variation in dengue virus. Trends Microbiol. 2000;8(2):74–7. doi: 10.1016/s0966-842x(99)01669-8 10664600

[89] Katzelnick LC, Gresh L, Halloran ME, Mercado JC, Kuan G, Gordon A, et al. Antibody-dependent enhancement of severe dengue disease in humans. Science. 2017;358(6365):929–32. doi: 10.1126/science.aan6836 29097492 PMC5858873

[90] Zompi S, Santich BH, Beatty PR, Harris E. Protection from secondary dengue virus infection in a mouse model reveals the role of serotype cross-reactive B and T cells. J Immunol. 2012;188(1):404–16. doi: 10.4049/jimmunol.1102124 22131327 PMC3244532

[91] Zhang C, Mammen MP Jr, Chinnawirotpisan P, Klungthong C, Rodpradit P, Monkongdee P, et al. Clade replacements in dengue virus serotypes 1 and 3 are associated with changing serotype prevalence. J Virol. 2005;79(24):15123–30. doi: 10.1128/JVI.79.24.15123-15130.2005 16306584 PMC1316048

[92] Gallichotte EN, Baric TJ, Nivarthi U, Delacruz MJ, Graham R, Widman DG, et al. Genetic Variation between Dengue Virus Type 4 Strains Impacts Human Antibody Binding and Neutralization. Cell Rep. 2018;25(5):1214–24. doi: 10.1016/j.celrep.2018.10.006 30380413 PMC6226424

[93] Visser I, Koenraadt CJM, Koopmans MPG, Rockx B. The significance of mosquito saliva in arbovirus transmission and pathogenesis in the vertebrate host. One Health. 2023;16:100506. doi: 10.1016/j.onehlt.2023.100506 37363242 PMC10288056

[94] Pingen M, Schmid MA, Harris E, McKimmie CS. Mosquito Biting Modulates Skin Response to Virus Infection. Trends Parasitol. 2017;33(8):645–57. doi: 10.1016/j.pt.2017.04.003 28495485

[95] Zamyatkin DF, Parra F, Alonso JMM, Harki DA, Peterson BR, Grochulski P, et al. Structural insights into mechanisms of catalysis and inhibition in Norwalk virus polymerase. J Biol Chem. 2008;283(12):7705–12. doi: 10.1074/jbc.M709563200 18184655

[96] Lee K, Kim D-E, Jang K-S, Kim S-J, Cho S, Kim C. Gemcitabine, a broad-spectrum antiviral drug, suppresses enterovirus infections through innate immunity induced by the inhibition of pyrimidine biosynthesis and nucleotide depletion. Oncotarget. 2017;8(70):115315–25. doi: 10.18632/oncotarget.23258 29383162 PMC5777774

[97] Chan KK, Wood BM, Fedorov AA, Fedorov EV, Imker HJ, Amyes TL, et al. Mechanism of the orotidine 5’-monophosphate decarboxylase-catalyzed reaction: evidence for substrate destabilization. Biochemistry. 2009;48(24):5518–31. doi: 10.1021/bi900623r 19435314 PMC2697262

[98] Donovan WP, Kushner SR. Purification and characterization of orotidine-5’-phosphate decarboxylase from Escherichia coli K-12. J Bacteriol. 1983;156(2):620–4. doi: 10.1128/jb.156.2.620-624.1983 6355062 PMC217875

[99] Rodaway S, Marcus A. In vivo synthesis of 6-azauridine 5’-triphosphate and incorporation of 6-azauridine into RNA of germinating wheat embryonic axes. Journal of Biological Chemistry. 1980;255(18):8402–4. doi: 10.1016/s0021-9258(18)43508-9 6157685

[100] Rada B, Dragún M. Antiviral action and selectivity of 6-azauridine. Ann N Y Acad Sci. 1977;284:410–7. doi: 10.1111/j.1749-6632.1977.tb21977.x 280143

[101] Migliaccio G, Tomassini JE, Carroll SS, Tomei L, Altamura S, Bhat B, et al. Characterization of resistance to non-obligate chain-terminating ribonucleoside analogs that inhibit hepatitis C virus replication in vitro. J Biol Chem. 2003;278(49):49164–70. doi: 10.1074/jbc.M305041200 12966103

[102] Nyström K, Trybala E, Said J, Roth A, Patzi Churqui M, Kärmander A, et al. Remdesivir is active in vitro against tick-borne encephalitis virus and selects for resistance mutations in the viral RNA-dependent RNA polymerase. Infect Dis (Auckl). 2025;0:1–8. doi: 10.1080/23744235.2025.2468510 39973341

[103] Kokic G, Hillen HS, Tegunov D, Dienemann C, Seitz F, Schmitzova J, et al. Mechanism of SARS-CoV-2 polymerase stalling by remdesivir. Nat Commun. 2021;12(1):279. doi: 10.1038/s41467-020-20542-0 33436624 PMC7804290

[104] Sanderson T, Hisner R, Donovan-Banfield I, Hartman H, Løchen A, Peacock TP, et al. A molnupiravir-associated mutational signature in global SARS-CoV-2 genomes. Nature. 2023;623(7987):594–600. doi: 10.1038/s41586-023-06649-6 37748513 PMC10651478

[105] Benarroch D, Egloff M-P, Mulard L, Guerreiro C, Romette J-L, Canard B. A structural basis for the inhibition of the NS5 dengue virus mRNA 2’-O-methyltransferase domain by ribavirin 5’-triphosphate. J Biol Chem. 2004;279(34):35638–43. doi: 10.1074/jbc.M400460200 15152003

[106] Dragoni F, Boccuto A, Picarazzi F, Giannini A, Giammarino F, Saladini F, et al. Evaluation of sofosbuvir activity and resistance profile against West Nile virus in vitro. Antiviral Res. 2020;175:104708. doi: 10.1016/j.antiviral.2020.104708 31931104

[107] Valdés JJ, Butterill PT, Růžek D. Flaviviridae viruses use a common molecular mechanism to escape nucleoside analogue inhibitors. Biochem Biophys Res Commun. 2017;492(4):652–8. doi: 10.1016/j.bbrc.2017.03.068 28322784

[108] Pierson TC, Sánchez MD, Puffer BA, Ahmed AA, Geiss BJ, Valentine LE, et al. A rapid and quantitative assay for measuring antibody-mediated neutralization of West Nile virus infection. Virology. 2006;346(1):53–65. doi: 10.1016/j.virol.2005.10.030 16325883

[109] Roy P, Walter Z, Berish L, Ramage H, McCullagh M. Motif-VI loop acts as a nucleotide valve in the West Nile Virus NS3 Helicase. Nucleic Acids Res. 2024;52(13):7447–64. doi: 10.1093/nar/gkae500 38884215 PMC11260461

[110] Jordheim LP, Durantel D, Zoulim F, Dumontet C. Advances in the development of nucleoside and nucleotide analogues for cancer and viral diseases. Nat Rev Drug Discov. 2013;12(6):447–64. doi: 10.1038/nrd4010 23722347

[111] Murakami E, Wang T, Babusis D, Lepist E-I, Sauer D, Park Y, et al. Metabolism and pharmacokinetics of the anti-hepatitis C virus nucleotide prodrug GS-6620. Antimicrob Agents Chemother. 2014;58(4):1943–51. doi: 10.1128/AAC.02350-13 24419340 PMC4023801

[112] Geutjes E-J, Tian S, Roepman P, Bernards R. Deoxycytidine kinase is overexpressed in poor outcome breast cancer and determines responsiveness to nucleoside analogs. Breast Cancer Res Treat. 2012;131(3):809–18. doi: 10.1007/s10549-011-1477-3 21465168

[113] Yue L, Saikawa Y, Ota K, Tanaka M, Nishimura R, Uehara T, et al. A functional single-nucleotide polymorphism in the human cytidine deaminase gene contributing to ara-C sensitivity. Pharmacogenetics. 2003;13(1):29–38. doi: 10.1097/00008571-200301000-00005 12544510

[114] Robins MJ, Peng Y, Damaraju VL, Mowles D, Barron G, Tackaberry T, et al. Improved syntheses of 5’-S-(2-aminoethyl)-6-N-(4-nitrobenzyl)-5’-thioadenosine (SAENTA), analogues, and fluorescent probe conjugates: analysis of cell-surface human equilibrative nucleoside transporter 1 (hENT1) levels for prediction of the antitumor efficacy of gemcitabine. J Med Chem. 2010;53(16):6040–53. doi: 10.1021/jm100432w 20718495

[115] Spratlin J, Sangha R, Glubrecht D, Dabbagh L, Young JD, Dumontet C, et al. The absence of human equilibrative nucleoside transporter 1 is associated with reduced survival in patients with gemcitabine-treated pancreas adenocarcinoma. Clin Cancer Res. 2004;10(20):6956–61. doi: 10.1158/1078-0432.CCR-04-0224 15501974

[116] Pastor-Anglada M, Cano-Soldado P, Molina-Arcas M, Lostao MP, Larráyoz I, Martínez-Picado J, et al. Cell entry and export of nucleoside analogues. Virus Res. 2005;107(2):151–64. doi: 10.1016/j.virusres.2004.11.005 15649561

[117] To EE. Cell and Tissue Specific Metabolism of Nucleoside and Nucleotide Drugs: Case Studies and Implications for Precision Medicine. Drug Metab Dispos. 2023;51(3):360–8. doi: 10.1124/dmd.122.000856 36446610

[118] Pan-Zhou X-R, Mayes BA, Rashidzadeh H, Gasparac R, Smith S, Bhadresa S, et al. Pharmacokinetics of IDX184, a liver-targeted oral prodrug of 2’-methylguanosine-5’-monophosphate, in the monkey and formulation optimization for human exposure. Eur J Drug Metab Pharmacokinet. 2016;41(5):567–74. doi: 10.1007/s13318-015-0267-4 25898809

[119] Zhao Y, Soh TS, Zheng J, Chan KWK, Phoo WW, Lee CC, et al. A crystal structure of the Dengue virus NS5 protein reveals a novel inter-domain interface essential for protein flexibility and virus replication. PLoS Pathog. 2015;11(3):e1004682. doi: 10.1371/journal.ppat.1004682 25775415 PMC4361662

[120] Lim SP, Noble CG, Seh CC, Soh TS, El Sahili A, Chan GKY, et al. Potent Allosteric Dengue Virus NS5 Polymerase Inhibitors: Mechanism of Action and Resistance Profiling. PLoS Pathog. 2016;12(8):e1005737. doi: 10.1371/journal.ppat.1005737 27500641 PMC4976923

[121] Brooks AJ, Johansson M, John AV, Xu Y, Jans DA, Vasudevan SG. The interdomain region of dengue NS5 protein that binds to the viral helicase NS3 contains independently functional importin beta 1 and importin alpha/beta-recognized nuclear localization signals. J Biol Chem. 2002;277(39):36399–407. doi: 10.1074/jbc.M204977200 12105224

[122] Khromykh AA, Sedlak PL, Westaway EG. trans-Complementation analysis of the flavivirus Kunjin ns5 gene reveals an essential role for translation of its N-terminal half in RNA replication. J Virol. 1999;73(11):9247–55. doi: 10.1128/JVI.73.11.9247-9255.1999 10516033 PMC112959

[123] Deatherage DE, Barrick JE. Identification of mutations in laboratory-evolved microbes from next-generation sequencing data using breseq. Methods Mol Biol. 2014;1151:165–88. doi: 10.1007/978-1-4939-0554-6_12 24838886 PMC4239701

