# Supplementary figures and images for: NS5-targeting nucleoside analogs inhibit dengue virus and other flaviviruses
Source: PLoS Pathog. 2026 Feb 17;22(2):e1013970. doi: 10.1371/journal.ppat.1013970 (PMC12928577; doi:10.1371/journal.ppat.1013970)

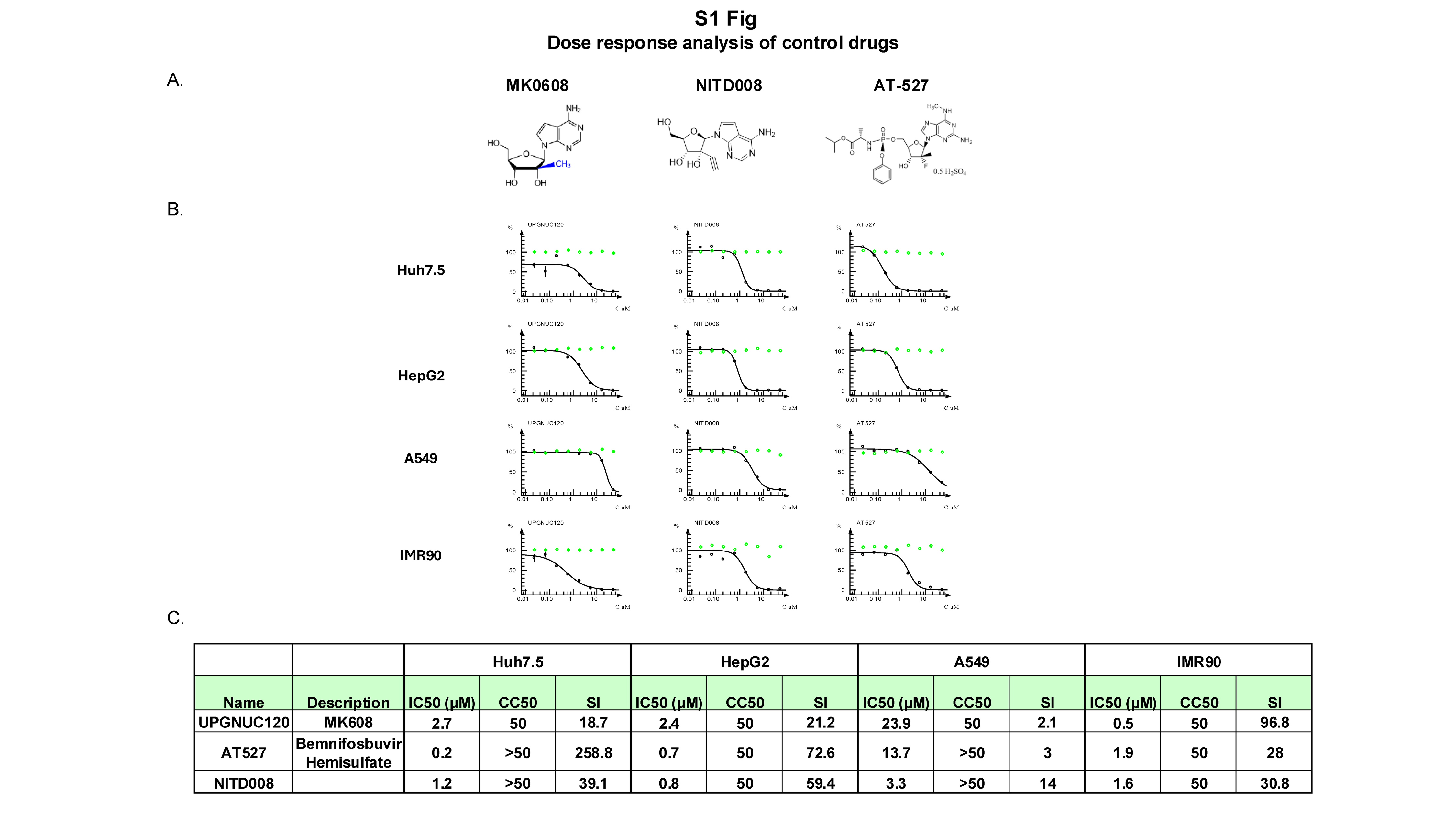

Supplement: S1 Fig — A. Chemical structures of control nucleoside analogs MK-0608, NITD008 and AT-527 B. Dose response analysis of MK0608, NITD008 and AT-527 in Huh7.5, HepG2, A549, and IMR90 cells infected with DENV2 at MOI = 2 (Huh7.5 and A549), MOI = 1 (HepG2), and MOI = 5 (IMR90). At 24 hours post-infection (hpi), viral antigen (4G2) and quantified via automated fluorescence microscopy and plotted as POC infection (black) and cell viability (green). C. Table of IC50, CC50, and SI values for control drugs tested in Huh7.5, HepG2, A549, and IMR90 cells infected with DENV2 as in B. SI shown for CC50 (ATPlite)/IC50. (TIF) [file ppat.1013970.s001.tif]

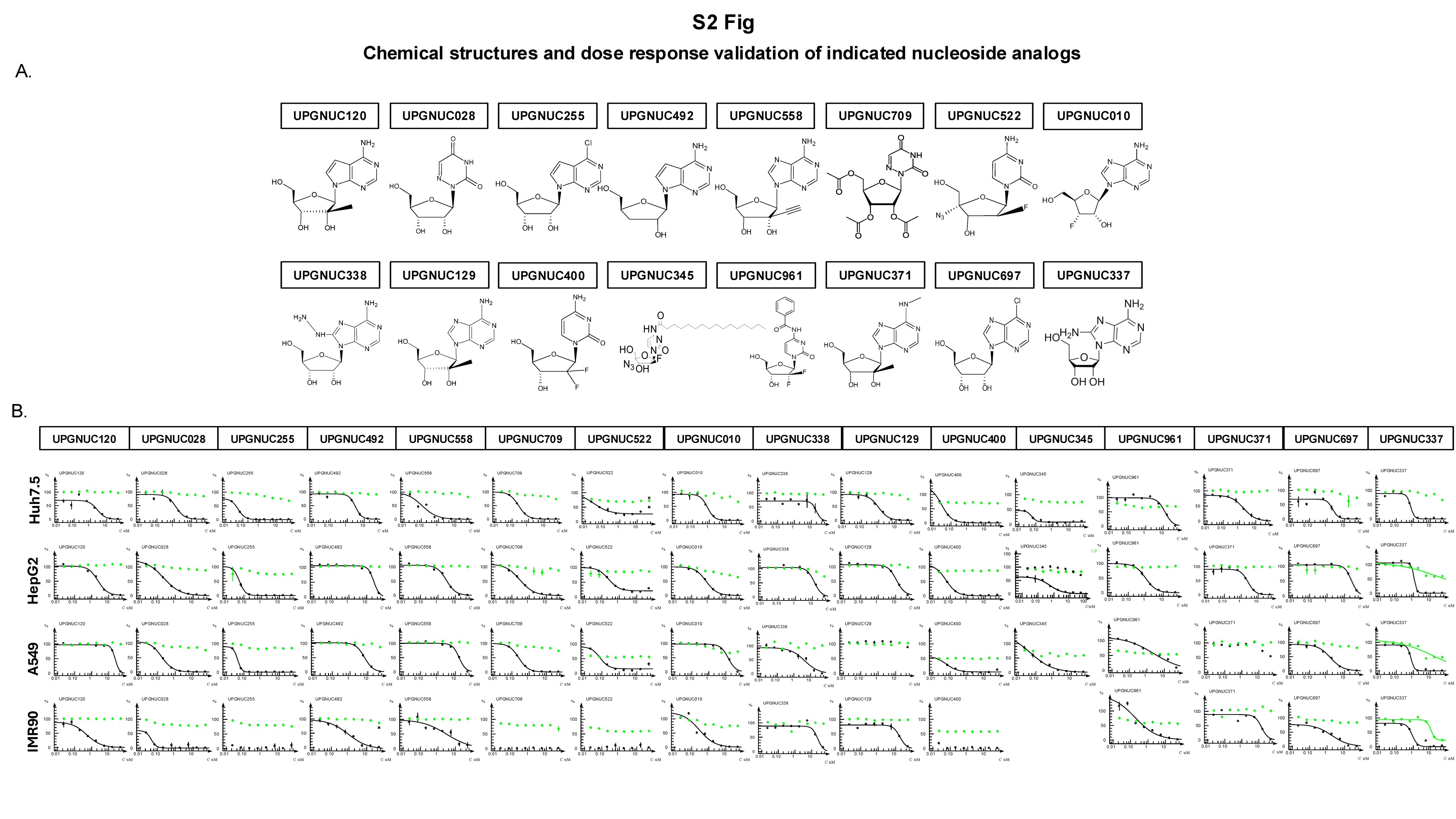

Supplement: S2 Fig — A. Chemical structures of 16 drugs selected from the primary screen having SI > 10 in at least one cell model (excluding Ribavirin, UPGNUC396). B. Dose response analysis of selected candidates in Huh7.5, HepG2, A549, and IMR90 cells against DENV2 infection. POC percent infection (black) POC cell viability (green). (TIF) [file ppat.1013970.s002.tif]

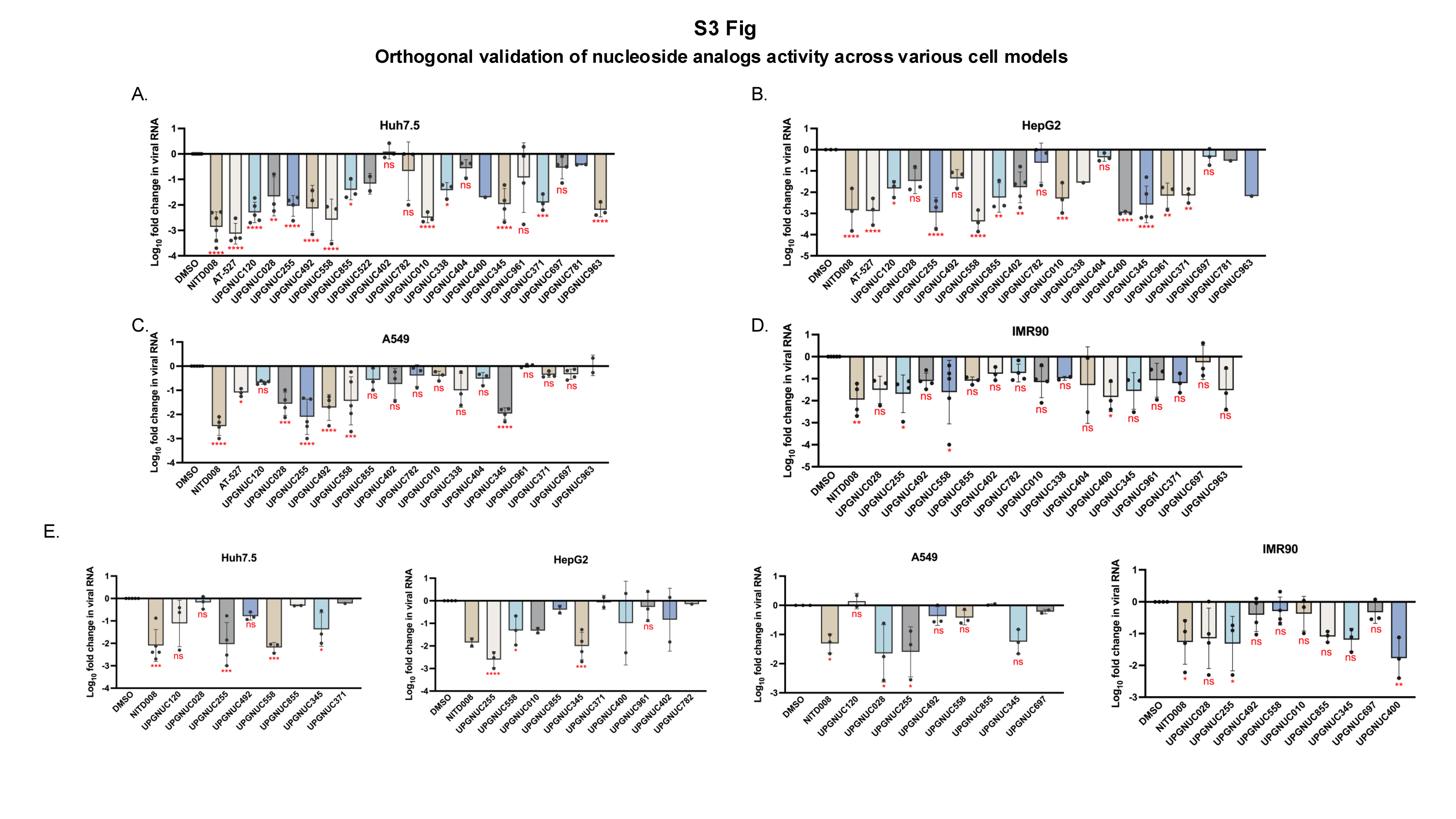

Supplement: S3 Fig — A-D.10 μM or E. 2 μM or DMSO vehicle control infected with DENV2 infection in Huh7.5 (MOI = 0.05), HepG2 (MOI = 0.5), A549 (MOI = 0.05), and IMR90 (MOI = 0.5) for 24 hpi, and subject to RT-qPCR. Data are presented as mean ± SD, showing viral RNA levels relative to the vehicle control (n ≥ 1–3 independent biological replicates). Statistical significance was determined on n ≥ 3 by one-way ANOVA with Dunnett’s correction for multiple comparisons on log10-transformed values (*P < 0.05, **P < 0.01, ***P < 0.001, ****P < 0.0001). (TIF) [file ppat.1013970.s003.tif]

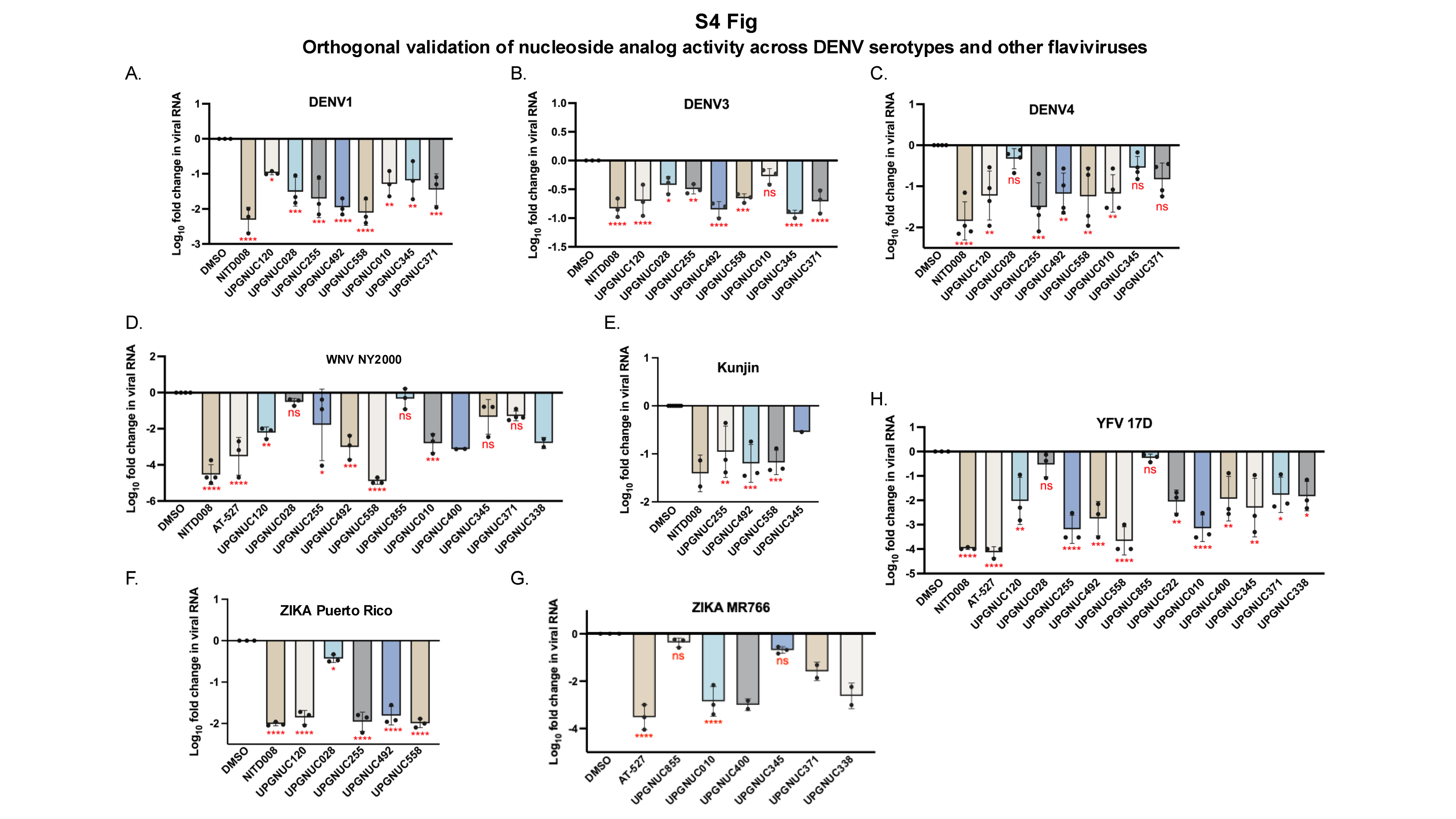

Supplement: S4 Fig — A. DENV1, B. DENV3 and C. DENV4 infection (MOI = 0.5 each, 24 hpi) in Huh7.5 cells pretreated with the indicated compounds (10 μM) or DMSO vehicle control. Data are presented as mean ± SD, showing viral RNA levels relative to the vehicle control (n ≥ 3 independent biological replicates). Statistical significance was determined for n ≥ 3 by one-way ANOVA with Dunnett’s correction for multiple comparisons on log10-transformed values (*P < 0.05, **P < 0.01, ***P < 0.001, ****P < 0.0001). qRT-PCR analysis of D. WNV infection E. KUNV infection F. ZIKA (Puerto Rico) infection G. ZIKA (MR766) infection and H. YFV (17D) infection at MOI = 0.5 each in Huh7.5 cells pretreated with the indicated compounds (10 μM) or DMSO vehicle control at 24 hpi. qPCR data are presented as mean ± SD, showing viral RNA levels relative to the vehicle control (n ≥ 1–3 independent biological replicates). Statistical significance was determined for n ≥ 3 by one-way ANOVA with Dunnett’s correction for multiple comparisons on log10-transformed values (*P < 0.05, **P < 0.01, ***P < 0.001, ****P < 0.0001). (TIF) [file ppat.1013970.s004.tif]

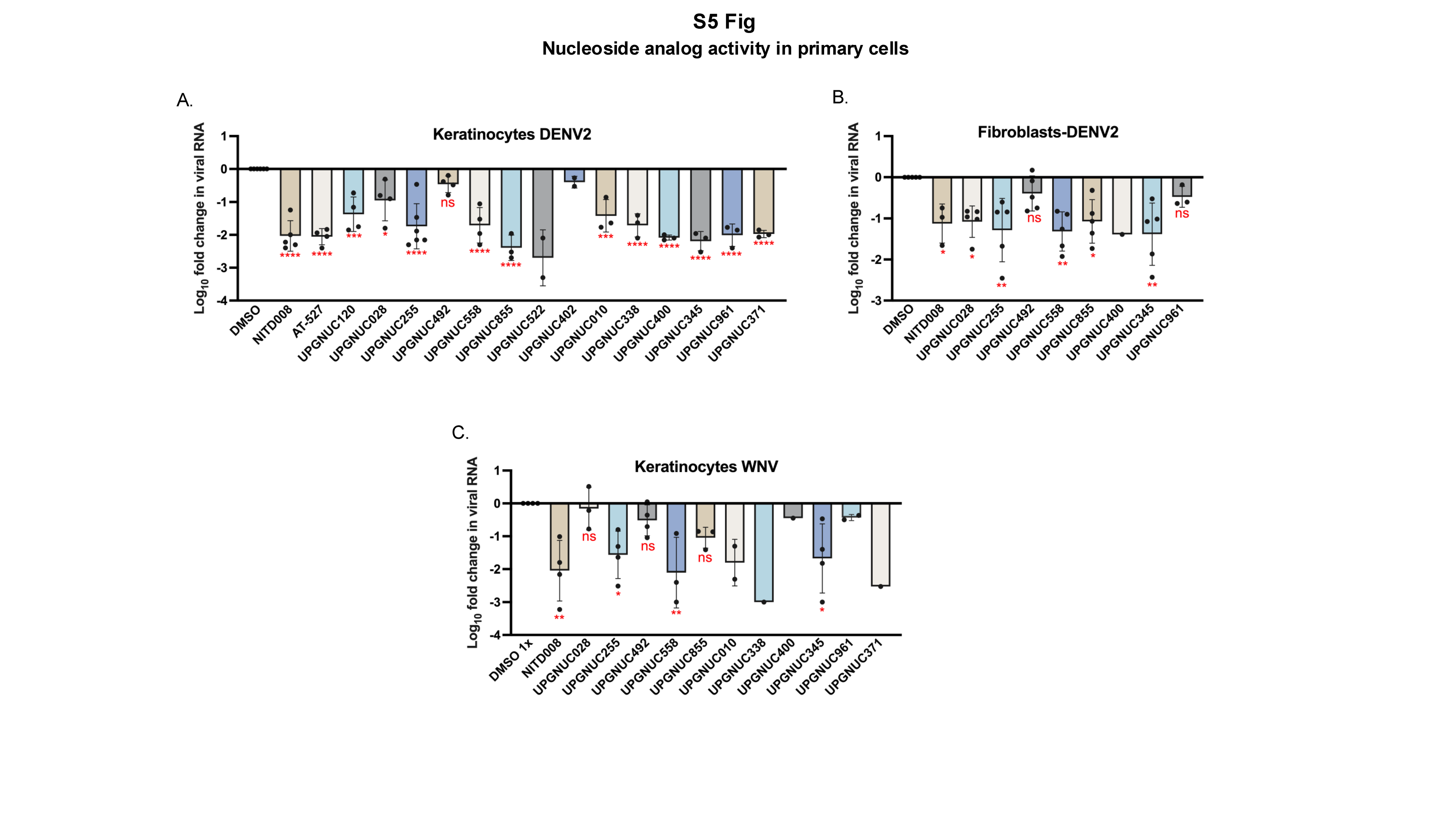

Supplement: S5 Fig — A. RT-qPCR analysis in primary keratinocytes pretreated with the indicated compounds (10 μM) or DMSO vehicle control were infected with DENV2 (MOI = 0.5) and subject to RT-qPCR 48hpi. B. Primary fibroblasts pretreated with the indicated compounds (10 μM) or DMSO vehicle control were infected with DENV2 (MOI = 0.5) and subject to RT-qPCR 48hpi. C. Primary keratinocytes pretreated with the indicated compounds (10 μM) or DMSO vehicle control were infected with WNV (MOI = 0.5) and subject to RT-qPCR 48hpi. For all qPCR, data are presented as mean ± SD, showing viral RNA levels relative to the vehicle control (n ≥ 1–3 independent biological replicates). Statistical significance was determined for n ≥ 3 by one-way ANOVA with Dunnett’s correction for multiple comparisons on log10transformed values (*P < 0.05, **P < 0.01, ***P < 0.001, ****P < 0.0001). (TIF) [file ppat.1013970.s005.tif]

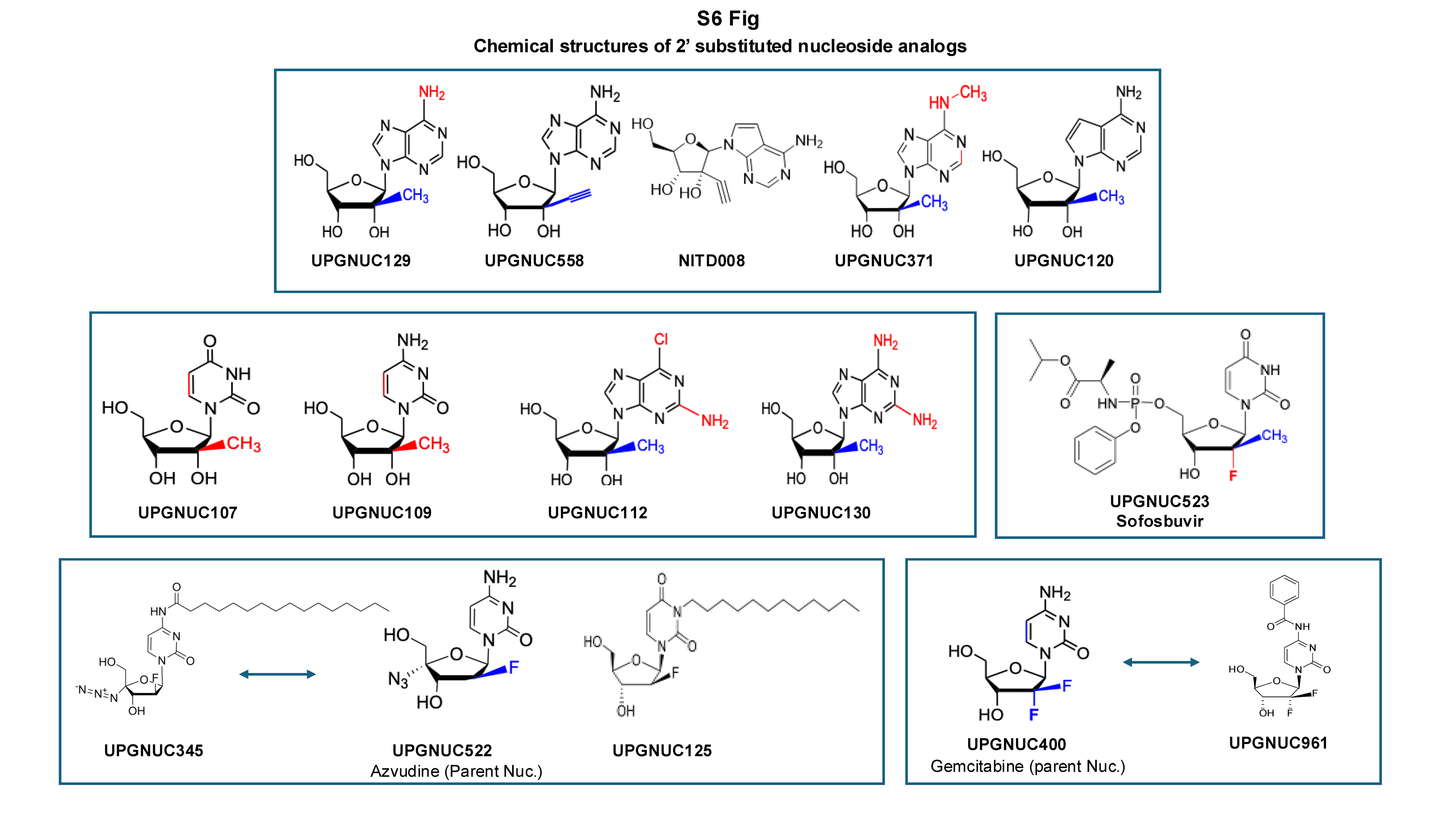

Supplement: S6 Fig — (TIF) [file ppat.1013970.s006.tif]

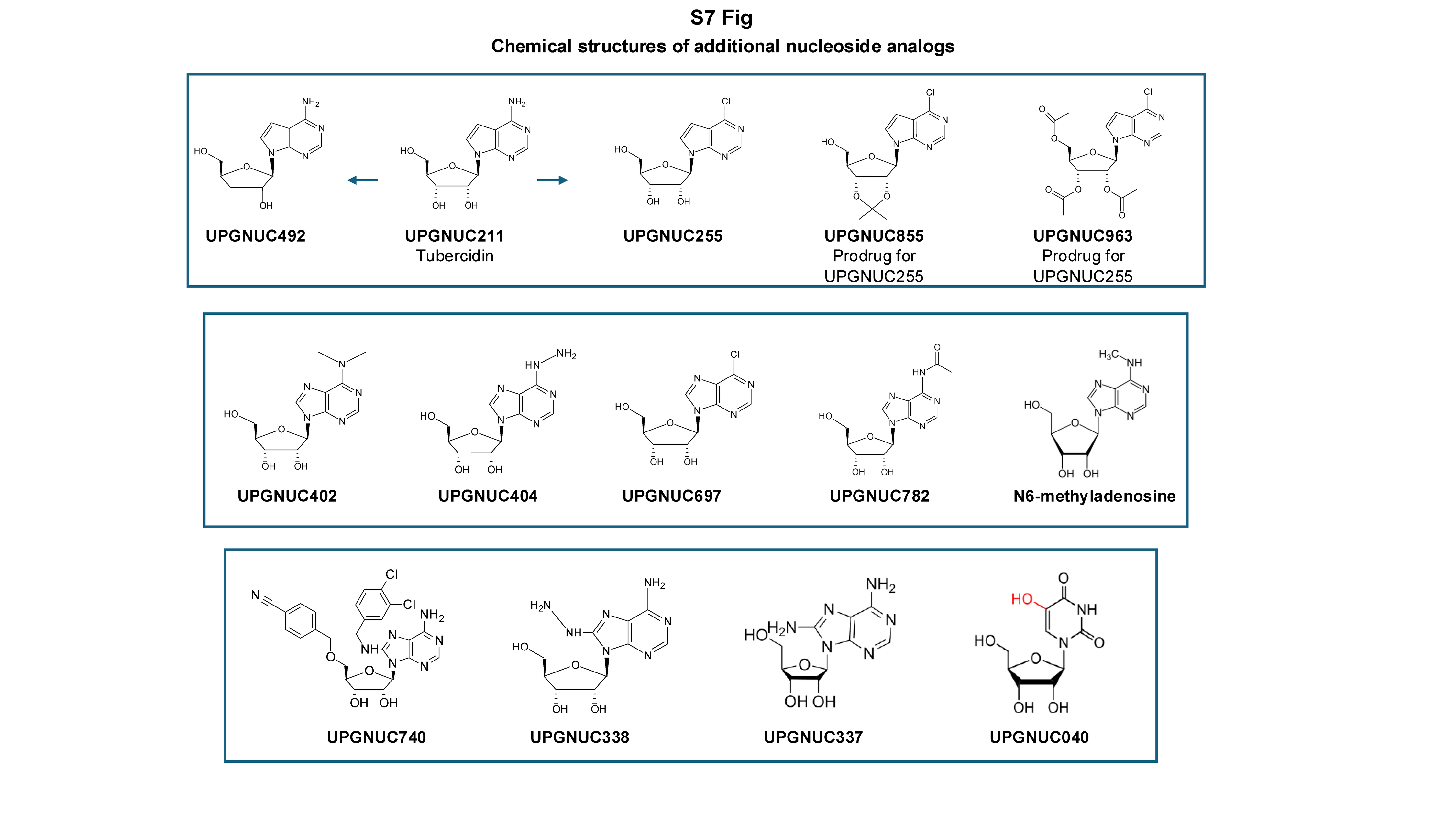

Supplement: S7 Fig — (TIF) [file ppat.1013970.s007.tif]

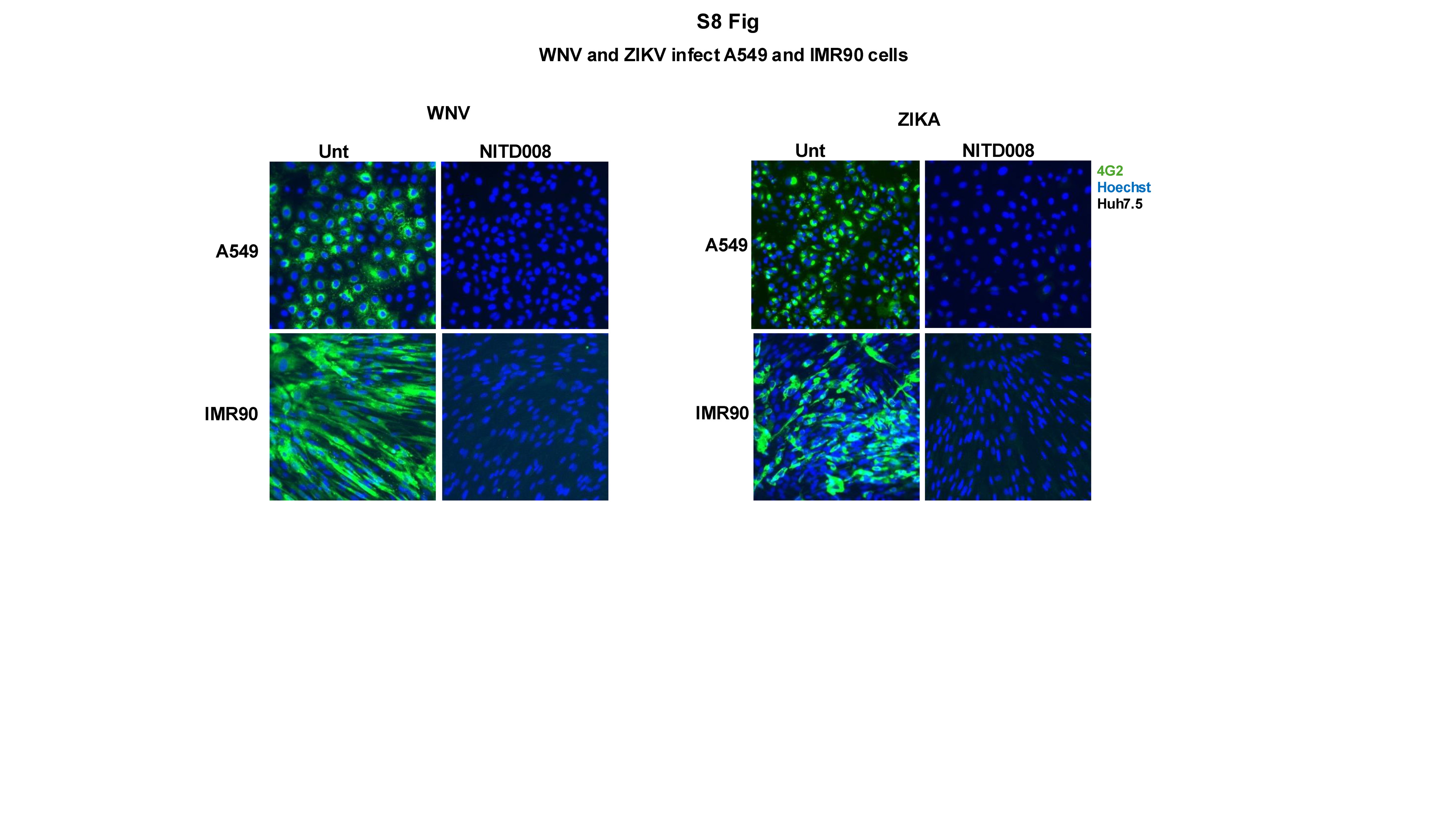

Supplement: S8 Fig — Representative microscopy images for WNV (NY2000) and ZIKA (MR766) infection in A549 and IMR90 cells. Cells were treated with either DMSO or NITD008 (10uM) and fixed 24 hpi and stained for viral infection (4G2, green) and cell number (Hoechst 33342, blue). 10 × magnification. (TIF) [file ppat.1013970.s008.tif]

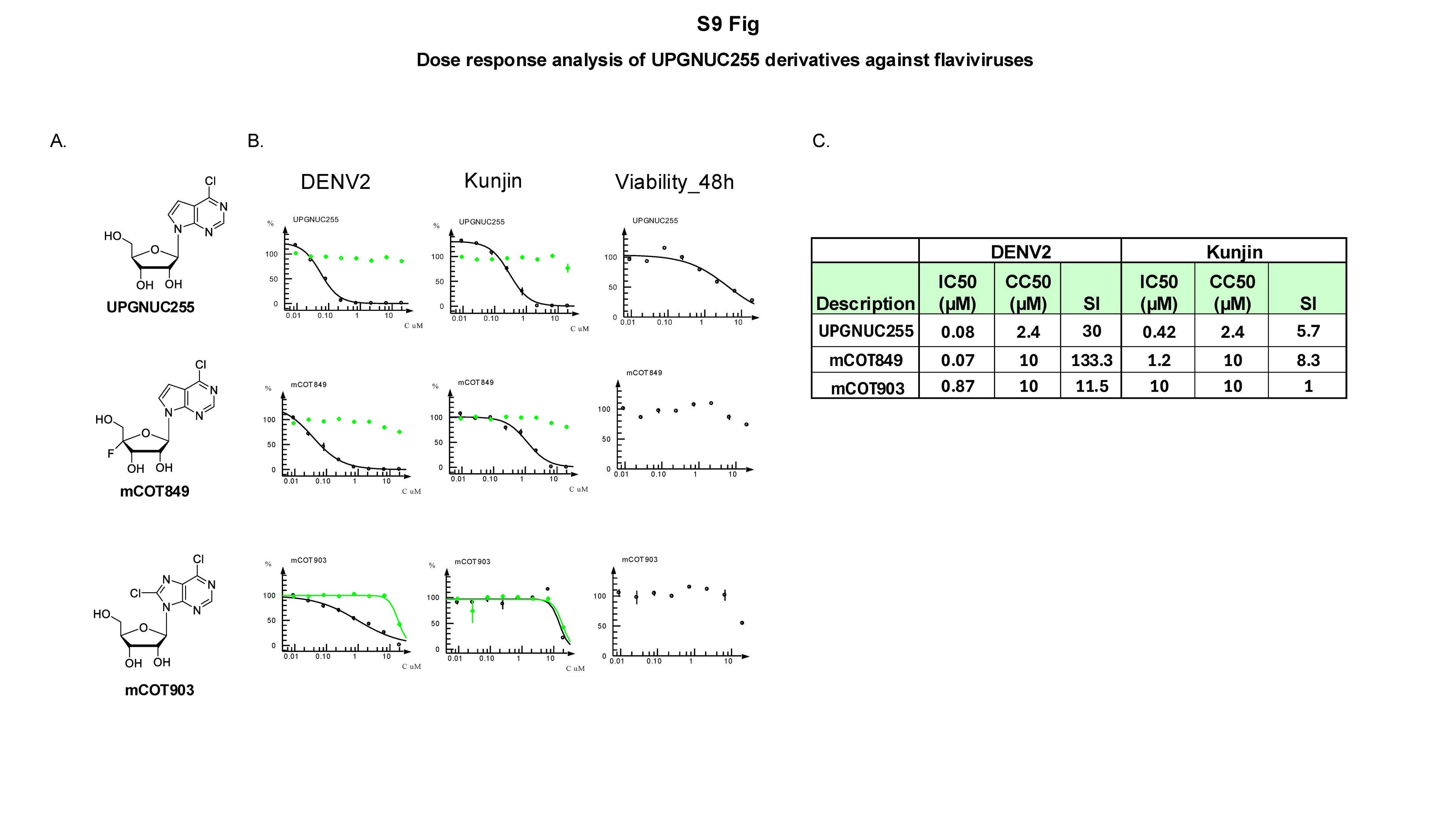

Supplement: S9 Fig — A. Chemical structures. B. Dose response analysis of indicated nucleoside analogs in Huh7.5 cells infected with DENV2 or KUNV for 24h and subject to automated microscopy or at 48h processed for cell viability (ATPlite). POC percent infection (black) POC cell viability (green). C. Table of IC50, CC50, and SI values for indicated drugs tested in Huh7.5 cells infected with DENV2 and KUNV. SI shown for CC50 (ATPlite)/IC50. (TIF) [file ppat.1013970.s009.tif]

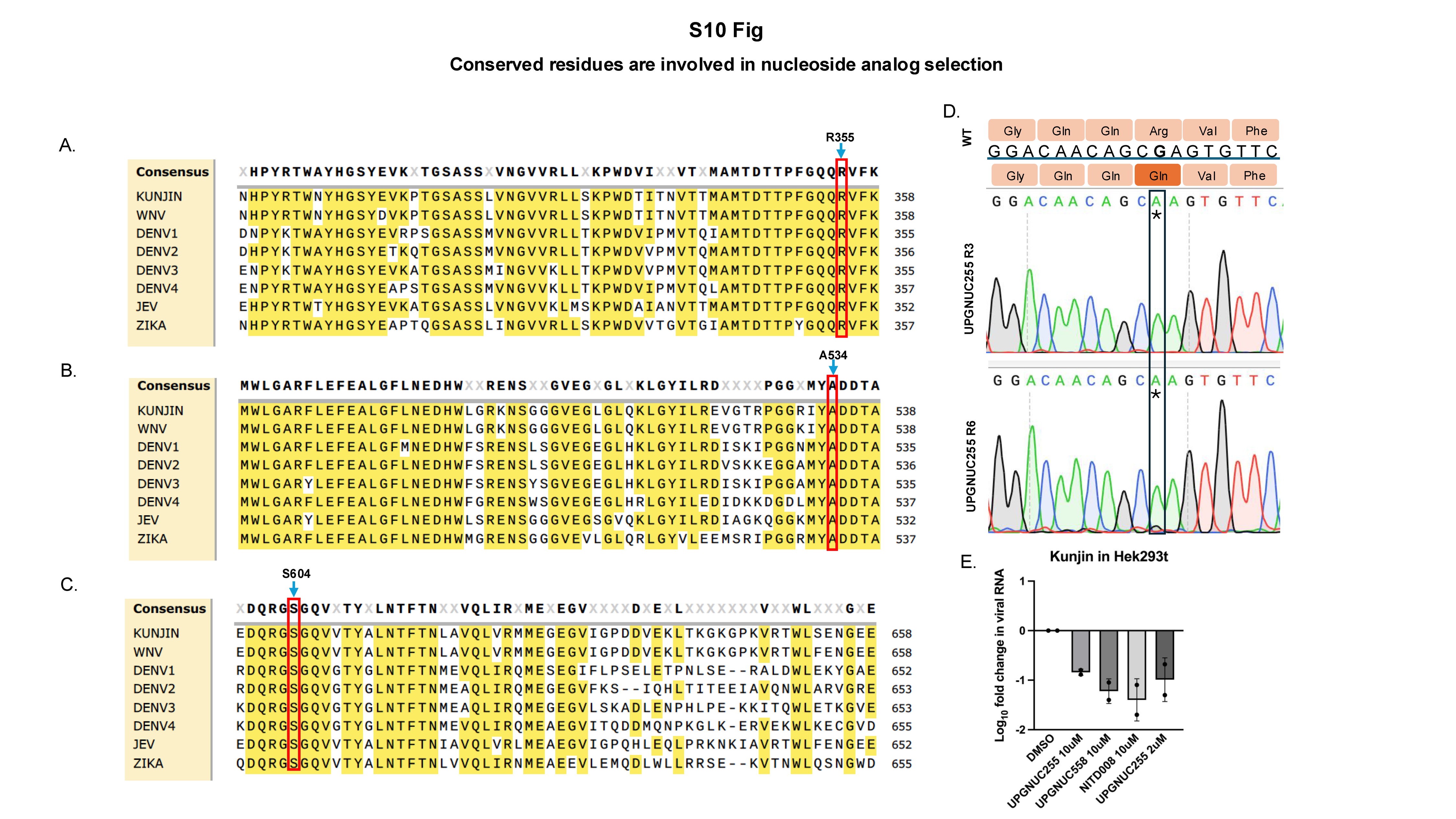

Supplement: S10 Fig — A-C. Alignment of sequences in the NS5 region of KUNV with indicated flaviviruses: Kunjin (AAP78942.1), West Nile (YP_001527887.1), DENV1 (QMW69322.1), DENV2 NGC (NP_739590.2), DENV3 (QUD37256.1), DENV4 (UCQ65264.1), Japanese encephalitis virus (JEV) (AIN36651.1), ZIKA Virus (AMQ34004.1). Conserved amino acids are highlighted in yellow and three amino acids A. R355, B. A534 and C. S604 are indicated in the red box. D. Traces of sanger sequencing are showing Kunjin NS5 RdRp gene mutation in UPGUC255_R3 and UPGNUC255_R6 resistant strains where Arginine-355 residue has been substituted by Glutamine (dark orange), *represented the substituted A in place of G. E. HEK293T cells were pretreated with the indicated nucleosides or vehicle were infected with KUNV (MOI = 0.5), and 24 hpi subject to RT-qPCR. Data are presented as mean ± SD, showing viral RNA levels relative to the vehicle control (n = 2). (TIF) [file ppat.1013970.s010.tif]
